# Improved Detection of Common Variants Associated with Schizophrenia and Bipolar Disorder Using Pleiotropy-Informed Conditional False Discovery Rate

**DOI:** 10.1371/journal.pgen.1003455

**Published:** 2013-04-25

**Authors:** Ole A. Andreassen, Wesley K. Thompson, Andrew J. Schork, Stephan Ripke, Morten Mattingsdal, John R. Kelsoe, Kenneth S. Kendler, Michael C. O'Donovan, Dan Rujescu, Thomas Werge, Pamela Sklar, The Psychiatric Genomics Consortium (PGC), Bipolar Disorder and Schizophrenia Working Groups, J. Cooper Roddey, Chi-Hua Chen, Linda McEvoy, Rahul S. Desikan, Srdjan Djurovic, Anders M. Dale

**Affiliations:** 1 KG Jebsen Centre for Psychosis Research, Institute of Clinical Medicine, University of Oslo, Oslo, Norway; 2 Division of Mental Health and Addiction, Oslo University Hospital, Oslo, Norway; 3 Department of Psychiatry, University of California San Diego, La Jolla, California, United States of America; 4 Multimodal Imaging Laboratory, University of California San Diego, La Jolla, California, United States of America; 5 Cognitive Sciences Graduate Program, University of California San Diego, La Jolla, California, United States of America; 6 Center for Human Development, University of California San Diego, La Jolla, California, United States of America; 7 Center for Human Genetic Research, Massachusetts General Hospital, Boston, Massachusetts, United States of America; 8 Virginia Institute for Psychiatric and Behavioral Genetics, Department of Psychiatry, Virginia Commonwealth University, Richmond, Virginia, United States of America; 9 MRC Centre for Neuropsychiatric Genetics and Genomics, School of Medicine, Cardiff University, Heath Park, Cardiff, United Kingdom; 10 Department of Psychiatry, University of Halle-Wittenberg, Halle, Germany; 11 Institute of Biological Psychiatry, MHC, Sct. Hans, University of Copenhagen, Copenhagen, Denmark; 12 The Division of Psychiatric Genetics and Genomics, Mount Sinai School of Medicine, New York, New York, United States of America; 13 Department of Radiology, University of California San Diego, La Jolla, California, United States of America; 14 Department of Neurosciences, University of California San Diego, La Jolla, California, United States of America; The University of Queensland, Australia

## Abstract

Several lines of evidence suggest that genome-wide association studies (GWAS) have the potential to explain more of the “missing heritability” of common complex phenotypes. However, reliable methods to identify a larger proportion of single nucleotide polymorphisms (SNPs) that impact disease risk are currently lacking. Here, we use a genetic pleiotropy-informed conditional false discovery rate (FDR) method on GWAS summary statistics data to identify new loci associated with schizophrenia (SCZ) and bipolar disorders (BD), two highly heritable disorders with significant missing heritability. Epidemiological and clinical evidence suggest similar disease characteristics and overlapping genes between SCZ and BD. Here, we computed conditional Q–Q curves of data from the Psychiatric Genome Consortium (SCZ; n = 9,379 cases and n = 7,736 controls; BD: n = 6,990 cases and n = 4,820 controls) to show enrichment of SNPs associated with SCZ as a function of association with BD and *vice versa* with a corresponding reduction in FDR. Applying the conditional FDR method, we identified 58 loci associated with SCZ and 35 loci associated with BD below the conditional FDR level of 0.05. Of these, 14 loci were associated with both SCZ and BD (conjunction FDR). Together, these findings show the feasibility of genetic pleiotropy-informed methods to improve gene discovery in SCZ and BD and indicate overlapping genetic mechanisms between these two disorders.

## Author Summary

Genome-wide association studies (GWAS) have thus far identified only a small fraction of the heritability of common complex disorders, such as severe mental disorders. We used a conditional false discovery rate approach for analysis of GWAS data, exploiting “genetic pleiotropy” to increase discovery of common gene variants associated with schizophrenia and bipolar disorders. Leveraging the increased power from combining GWAS of two associated phenotypes, we found a striking overlap in polygenic signals, allowing for the discovery of several new common gene variants associated with bipolar disorder and schizophrenia that were not identified in the original analysis using traditional GWAS methods. Some of the gene variants have been identified in other studies with large targeted replication samples, validating the present findings. Our pleiotropy-informed method may be of significant importance for detecting effects that are below the traditional genome-wide significance level in GWAS, particularly in highly polygenic, complex phenotypes, such as schizophrenia and bipolar disorder, where most of the genetic signal is missing (i.e., “missing heritability”). The findings also offer insights into mechanistic relationships between bipolar disorder and schizophrenia pathogenesis.

## Introduction

Converging evidence suggests that complex human phenotypes are influenced by numerous genes each explaining a small proportion of the variance [1]. Though thousands of single nucleotide polymorphisms (SNPs) have been identified by genome-wide association studies (GWAS) [2], [3], these SNPs fail to explain a large proportion of the heritability of most complex phenotypes studied. This is commonly referred to as the ‘missing heritability’ problem. Recent findings indicate that GWAS have the potential to explain a greater proportion of the heritability of common complex phenotypes [4]–[6], and more SNPs are likely to be identified in larger samples [7]. Due to the polygenic nature of most complex traits and disorders, a large number of SNPs are likely to have associations too small in magnitude to be identified with currently available sample sizes [8]. New analytical methods are therefore needed to reliably identify a larger proportion of SNPs associated with complex diseases and phenotypes, since recruitment and genotyping of sufficiently large samples for existing methods may be impractical and prohibitively expensive. Genetic pleiotropy is defined as a single gene or variant being associated with more than one distinct phenotype. In the present study we use a new genetic pleiotropy-informed approach for GWAS to capture more of the polygenic effects in complex phenotypes. Given the high number of traits in humans, and the relatively small number of genes (∼20,000), some genes have to affect multiple traits (genetic pleiotropy) [10]. By combining independent GWAS from associated disorders, we hypothesize that for disorders with related etiologies a genetic pleiotropy-informed approach can significantly improve gene discovery and help capture more of the missing heritability.

Recent findings suggest overlapping SNPs between several human traits [9], [11] and disorders [12]–[14]. To date, methods to assess this genetic pleiotropy have not taken full advantage of the existing GWAS data and the majority of studies have focused on the subset of SNPs exceeding a Bonferroni-corrected threshold of significance for each trait or disorder [12]–[14]. However, this approach cannot detect SNPs that only reach genome-wide significance in the combined analysis but do not meet Bonferroni-corrected significance in the individual phenotype (hereafter referred to as polygenic pleiotropy). Combining GWAS statistics from two disorders also provides increased power to discover genes associated with common biological mechanisms, and thus inform on overlapping pathophysiological relationships between the disorders. In the current study, we use a pleiotropy-informed statistical approach to improve gene discovery in schizophrenia and bipolar disorder, two disorders with high heritability [15], where most of the underlying genetic architecture remains unknown [13], [14], despite recent discoveries [13], [14], [16], [17].

Schizophrenia and bipolar disorder share several clinical characteristics [18]–[20], including psychotic symptoms, disorders of thought and impairment of cognitive functions [21]. The disorders are often also treated with similar pharmacological agents [18], [19]. Whether schizophrenia and bipolar disorder should be regarded as separable disease entities or as a single disease with a spectrum of symptoms [18]–[20], as proposed in the continuum hypothesis of psychosis [22], has been much discussed. With the forthcoming revision of the Diagnostic and Statistical Manual of Mental Disorders (DSM), this question has received renewed attention [19], [20], [23]. Both disorders have an estimated heritability of 0.7–0.8, and are regarded as complex disorders with a polygenic architecture. Several lines of evidence have suggested overlapping genetic susceptibility in bipolar disorder and schizophrenia [15], [24]–[26]. Recently, a combined analysis of two large GWAS (16,374 cases and 12,044 controls) revealed three loci (*CACNA1C*, rs4765905, *p* = 7.0×10^−9^, *ANK3* rs10994359, *p* = 2.5×10^−8^, *ITIH3/4* region rs2239547, *p* = 7.8×10^−9^) significantly associated with both disorders (Fisher's combined p in combined samples) [13], [14]. Still, given the high degree of heritability and large similarities in clinical phenotypes, there are likely several more undiscovered overlapping genetic factors.

Here, using summary statistics from two independent large GWAS, we applied a model-free statistical analysis method to identify SNPs exhibiting pleiotropic relationships between schizophrenia and bipolar disorder. First, we separated out the common controls in the bipolar disorder and schizophrenia samples [13], [14], ensuring non-overlapping samples. After applying genomic inflation control, we computed the conditional empirical cumulative distribution functions (cdfs) of the corrected p-values. Empirical cdfs for schizophrenia SNP p-values were determined conditional on the significance of the corresponding nominal p-values in bipolar disorder, and *vice versa*. For each nominal p-value, an estimate of the conditional False Discovery Rate (FDR) was obtained from the conditional empirical cdfs [5]. Using this conditional FDR method, we constructed two-dimensional FDR “look-up” tables, with FDR in schizophrenia SNPs computed conditional on nominal bipolar disorder p-values, and *vice versa*. Using these tables we identified 58 loci associated with schizophrenia and 35 loci associated with bipolar disorder at a conditional FDR level of 0.05. We used a *conjunction* method to investigate SNPs significantly associated with both schizophrenia and bipolar disorder. Specifically, we computed the conditional FDR for schizophrenia given bipolar disorder nominal p-values, and conditional FDR for bipolar disorder given schizophrenia nominal p-values, and took the maximum of both values as the conjunction FDR. With this approach we identified 14 pleiotropic loci indicating several overlapping genetic risk factors for the two disorders. Finally, using mixture model-based analyses we estimated the proportion and distribution of non-null SNPs, demonstrating that the large increase in power from using conditional vs. unconditional FDR methods is derived from the high polygenicity of both phenotypes with many test statistics just below significance thresholds, and the largely overlapping distribution (high degree of pleiotropy) of non-null SNPs for schizophrenia and bipolar disorder.

## Results

### Q–Q plots of schizophrenia SNPs conditional on association with bipolar disorder and vice versa

Under large-scale testing paradigms, such as GWAS, quantitative estimates of likely true associations can be estimated from distributions of summary statistics [27], [28]. A common method for visualizing the ‘enrichment’ of statistical association relative to that expected under the global null hypothesis is through Q-Q plots of nominal p-values obtained from GWAS summary statistics. The usual Q-Q curve has the nominal p-value, denoted by “p”, as the y-ordinate and the corresponding value of the empirical cdf, here denoted by “q,” as the x-ordinate. Under the global null hypothesis the theoretical distribution is uniform on the interval [0,1]. As is common in GWAS, we instead plot −log_10_ p against −log_10_ q to emphasize tail probabilities of the theoretical and empirical distributions. As such, genetic ‘enrichment’ refers to a leftward shift in the Q-Q curve, corresponding to a larger fraction of SNPs with nominal −log_10_ p-value greater than or equal to a given threshold. *Conditional* Q-Q plots are formed by creating subsets of SNPs based on values of an additional variable (auxiliary measure) for each SNP, and computing Q-Q plots separately for each subset of SNPs. If SNP enrichment is captured by variation in the auxiliary measure, this is expressed as successive leftward deflections in conditional Q-Q plots as values of the additional variable increase.

**Figure 1:**
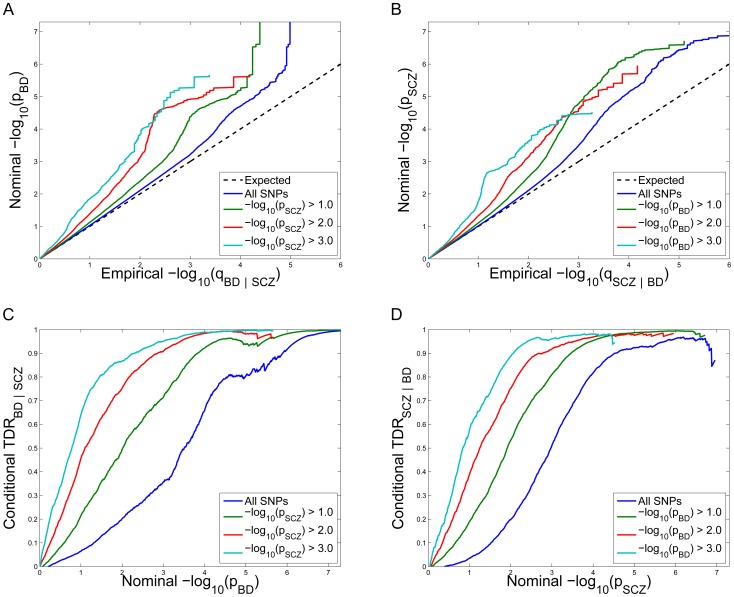
Stratified Q–Q plot and Stratified True Discovery Rate plots. *Upper panel:* Stratified Q-Q plot of nominal versus empirical −log_10_ p-values (corrected for inflation) in A) schizophrenia (SCZ) below the standard GWAS threshold of p<5×10^−8^ as a function of significance of the association with bipolar disorder (BD) at the level of −log_10_(p)>0, −log_10_(p)>1, −log_10_(p)>2, −log_10_(p)>3 corresponding to p<1, p<0.1, p<0.01, p<0.001, respectively, and in B) BD below the standard GWAS threshold of p<5×10^−8^ as a function of significance of association with SCZ at the level of −log_10_(p)>0, −log_10_(p)>1, −log_10_(p)>2, −log_10_(p)>3 corresponding to p<1, p<0.1, p<0.01, p<0.001, respectively. Dotted lines indicate the null-hypothesis. *Lower panel:* Stratified True Discovery Rate (TDR) plots illustrating the increase in TDR associated with increased pleiotropic enrichment in C) SCZ conditional on nominal BD p-values (SCZ|BD), and D) BD conditional on nominal SCZ p-values (BD|SCZ). For more information about QQ plots, see Text S1.

Conditional Q-Q plots for schizophrenia given nominal p-values of association with bipolar disorder (SCZ|BD; Figure 1A) show enrichment across different levels of significance for bipolar disorder. The earlier departure from the null line (leftward shift) suggests a greater proportion of true associations for a given nominal schizophrenia p-value. Successive leftward shifts for decreasing nominal bipolar disorder p-value thresholds indicate that the proportion of non-null effects in schizophrenia varies considerably across different levels of association with bipolar disorder. For example, the proportion of SNPs in the −log_10_(p_BD_) ≥3 category reaching a given significance level for schizophrenia (e.g., −log_10_(p_SCZ_) ≥4) is roughly 50 times greater than for the −log_10_(p_BD_) ≥0 category (all SNPs), indicating a high level of enrichment. An even stronger pleiotropic enrichment can be seen for bipolar disorder conditioned on nominal p-values of association with schizophrenia (BD|SCZ; Figure 1B), Here, the proportion of SNPs in the −log_10_(p_SCZ_) ≥3 category reaching a given significance level for bipolar disorder (e.g., −log_10_(p_BD_)≥4) is roughly 500 times greater than for the −log_10_(p_SCZ_)≥0 category (all SNPs), indicating a very high level of enrichment.

#### Conditional True Discovery Rate (TDR) in schizophrenia is increased by bipolar disorder, and vice versa

Since categories of SNPs with stronger pleiotropic enrichment are more likely to be associated with schizophrenia, to maximize power for discovery all tag SNPs should not be treated exchangeably. Specifically, variation in enrichment across pleiotropic categories is expected to be associated with corresponding variation in the TDR (equivalent to 1-FDR) [29] for association of SNPs with schizophrenia. A conservative estimate of the TDR for each nominal p-value is equivalent to 1 – (p/q), easily read off from conditional Q-Q plots (see Material and Methods). This relationship is shown for schizophrenia conditioned on nominal bipolar disorder p-values (SCZ|BD; Figure 1C) and bipolar disorder conditioned on nominal schizophrenia p-values (BD|SCZ; Figure 1D). For a given conditional TDR the corresponding estimated nominal p-value threshold varies with a factor of 100 from the most to the least enriched SNP category for schizophrenia conditioned on bipolar disorder (SCZ|BD), and approximately a factor of 500 for bipolar disorder conditioned on schizophrenia (BD|SCZ).

### Schizophrenia gene loci identified with conditional FDR

**Figure 2:**
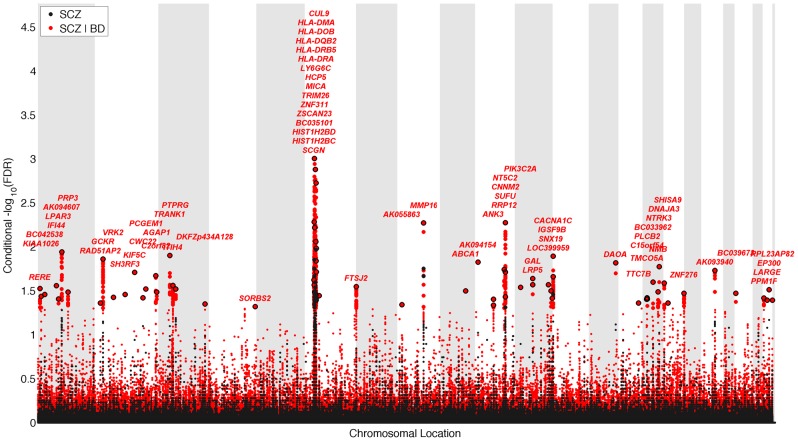
“Conditional Manhattan plot” of conditional −log_10_ (FDR) values for schizophrenia (SCZ) alone (black) and SCZ given bipolar disorder (BD; SCZ|BD, red). SNPs with conditional −log_10_ FDR>1.3 (i.e. FDR<0.05) are shown with large points. A black line around the large points indicates the most significant SNP in each LD block and this SNP was annotated with the closest gene, which is listed above the symbols in each locus. The figure shows the localization of significant loci. Details about the loci are provided in Table 1.

We constructed a “conditional” Manhattan plot for schizophrenia showing the FDR conditional on bipolar disorder (Figure 2) and identified significant loci on a total of 18 chromosomes (1–4, 6–16, 18, 20 and 22) associated with schizophrenia leveraging the reduced FDR obtained by the associated bipolar disorder phenotype. To estimate the number of independent loci, we ‘pruned’ the associated SNPs (removed SNPs with linkage disequilibrium (LD)>0.2), and identified a total of 58 independent loci with a significance threshold of conditional FDR<0.05 (Table 1). Using the more conservative conditional FDR threshold of 0.01, 9 independent loci remained significant. One locus was located in the HLA region on chromosome 6. Of note, using a standard Bonferroni-corrected approach, no loci would have been discovered. Using the FDR method in schizophrenia alone, 4 loci were identified. Of these, the regions close to *TRIM26* (6p21.3), *MMP16* (8q21.3) and *NT5C2* (10q24.32) have been identified in earlier GWAS studies after including large replication samples [13]. The remaining loci would not have been identified in the current sample without using the pleiotropy-informed conditional FDR method. Of interest, the *VRK2* region (2p16.1) was identified in the previous sample after including a large schizophrenia replication sample [30], and the *ITIH4* region (3p21.1), *ANK3* (10q21) and *CACNA1C* (12p13.3) were discovered previously in the same, combined schizophrenia and bipolar disorder sample [13], [14]. Thus, the current pleiotropy-informed FDR method validated 7 loci discovered in considerably larger samples, and discovered 51 new loci.

**Table 1:** Conditional FDR; SCZ loci given BD (SCZ|BD).

Locus	SNP	neighbor gene	chr	pval SCZ	fdr SCZ	fdr SCZ|BD
1	rs2252865	*RERE*	*1p36.23*	4.76E-04	0.377	0.030
2	rs11579756	*KIAA1026*	*1p36.21*	1.17E-04	0.203	0.037
3	rs4949526	*BC042538*	*1p35.2*	1.11E-04	0.181	0.035
4	rs4650608	*IFI44*	*1p31.1*	2.06E-04	0.257	0.028
5	rs4907103	*LPAR3*	*1p22.3*	9.77E-05	0.181	0.039
6	rs1625579	*AK094607*	*1p21.3*	3.76E-06	0.065	0.011
7	rs11205362	*PRP3*	*1q21.1*	1.11E-03	0.489	0.033
8	rs10495658	*RAD51AP2*	*2p24.2*	.3.99E-05	0.115	0.044
9	rs813592	*GCKR*	*2p23*	2.71E-05	0,095	0.014
10	rs10189138	*VRK2* †	*2p16.1*	1.42E-04	0.229	0.038
11	rs11692886	*SH3RF3*	*2q13*	1.05E-04	0.181	0.035
12	rs6435387	*KIF5C*	*2q23.1*	4.28E-05	0.115	0.020
13	rs17180327	*CWC22*	*2q31.3*	1.29E-05	0.080	0.038
14	rs17662626	*PCGEM1*	*2q32*	7.79E-05	0.161	0.030
15	rs2675968	*C2orf82*	*2q37.1*	5-64E-05	0-143	0-021
16	rs4663627	*AGAP1*	*2q37*	1.31E-04	0.203	0.033
17	rs13072940	*TRANK1*	*3p22.2*	1.27E-05	0.080	0.013
18	rs4687657	*ITIH4* †	*3p21.1*	1.56E-04	0.229	0.028
19	rs11130874	*PTPRG*	*3p21-p14*	9.45E-06	0.077	0.030
20	rs9838229	*DKFZp434A128*	*3q26.33*	2.89E-05	0.104	0.045
21	rs13150700	*SORBS2*	*4q35.1*	2.77E-04	0.286	0.048
22	rs9379780	*SCGN*	*6p22.3-p22.1*	3.78E-06	0.065	0.024
	rs198829	*HIST1H2BC*	*6p22.1*	2.18E-05	0.088	0.027
23	rs7749823	*HIST1H2BD*	*6p21.3*	1.32E-07	**0.014**	0.005
	rs17693963	*BC035101*	*6p22.1*	1.87E-07	**0.022**	0.001
	rs13190937	*ZSCAN23*	*6p22.1*	1.23E-04	0.203	0.033
	rs3130893	*ZNF311*	*6p22.1*	3.83E-06	0.065	0.006
	rs2523722	*TRIM26* †	*6p21.32-p22.1*	2.54E-07	**0.025**	0.001
	rs2596565	*MICA*	*6p21.33*	9.33E-06	0.077	0.009
	rs2284178	*HCP5*	*6p21.3*	3.31E-04	0.316	0.036
	rs805294	*LY6G6C*	*6p21.33*	1.11E-04	0.181	0.039
	rs9268858	*HLA-DRA*	*6p21.3*	1.66E-05	0.084	0.041
	rs9268862	*HLA-DRA*	*6p21.3*	6.21E-07	**0.037**	0.002
	rs502771	*HLA-DRB5*	*6p21.3*	2.97E-05	0.104	0.039
	rs9276601	*HLA-DQB2*	*6p21*	3.07E-05	0.104	0.015
	rs7383287	*HLA-DOB*	*6p21.3*	2.71E-05	0.095	0.019
	rs1480380	*HLA-DMA*	*6p21.3*	1.06E-05	0.077	0.010
24	rs9462875	*CUL9*	*6p21.1*	1.61E-04	0.229	0.036
25	rs7787274	*FTSJ2*	*7p22*	3.27E-04	0.316	0.028
26	rs12543276	*AK055863*	*8p23.1*	1.38E-04	0.203	0.046
27	rs7004633	*MMP16* †	*8q21.3*	1.70E-07	**0.018**	0.005
28	rs2254884	*ABCA1*	*9q31.1*	1.17E-04	0.203	0.032
29	rs6602217	*AK094154*	*10p14*	2.29E-05	0.095	0.015
30	rs7084499	*ANK3* †	*10q21*	1.74E-04	0.229	0.040
31	rs2153522	*ANK3* †	*10q21*	7.92E-04	0.449	0.046
32	rs7895695	*RRP12*	*10q24.1*	3.57E-05	0.115	0.018
33	rs2298278	*SUFU*	*10q24.32*	1.24E-03	0.527	0.037
	rs10883817	*CNNM2*	*10q24.32*	1.13E-05	0.080	0.020
	rs11191580	*NT5C2* †	*10q24.32*	1.71E-06	**0.049**	0.005
34	rs4356203	*PIK3C2A*	*11p15.5-p14*	5.48E-05	0.128	0.029
35	rs676318	*LRP5*	*11q13.4*	1.41E-05	0.080	0.023
36	rs6591348	*GAL*	*11q13.3*	1.16E-05	0.080	0.027
37	rs17126243	*LOC399959*	*11q24.1*	1.29E-05	0.080	0.027
38	rs11222395	*SNX19*	*11q25*	1.36E-04	0.203	0.032
39	rs7106715	*IGSF9B*	*11q25*	6.52E-05	0.143	0.039
40	rs7972947	*CACNA1C* †	*12p13.3*	5.32E-07	**0.035**	0.013
41	rs1006737	*CACNA1C*	*12p13.3*	3.52E-05	0.104	0.022
42	rs4517638	*DAOA*	*13q34*	1.10E-05	0.077	0.015
43	rs961196	*TTC7B*	*14q32.11*	3.07E-03	0.662	0.044
44	rs1502404	*TMCO5A*	*15q14*	1.04E-03	0.489	0.040
45	rs724729	*C15orf54*	*15q14*	4.70E-05	0.128	0.038
46	rs1869901	*PLCB2*	*15q15*	2.03E-04	0.257	0.039
47	rs2414718	*BC033962*	*15q22.2*	4.59E-05	0.128	0.025
48	rs1051168	*NMB*	*15q22*	1.27E-04	0.203	0.033
49	rs1078163	*NTRK3*	*15q25*	2.67E-05	0.095	0.017
50	rs2304634	*DNAJA3*	*16p13.3*	7.90E-05	0.161	0.026
51	rs12708772	*SHISA9*	*16p13.12*	3.12E-03	0.662	0.044
52	rs4785714	*ZNF276*	*16q24.3*	1.34E-03	0.527	0.034
53	rs12966547	*AK093940*	*18q21.2*	6.23E-06	0.071	0.019
54	rs159788	*BC039673*	*20p13*	1.23E-03	0.527	0.034
55	rs381523	*PPM1F*	*22q11.22*	1.55E-03	0.560	0.038
56	rs9621795	*LARGE*	*22q12.3*	1.66E-05	0.084	0.041
57	rs5758209	*EP300*	*22q13.2*	5.06E-06	0.068	0.031
58	rs28729663	*RPL23AP82*	*22q13.33*	1.82E-04	0.257	0.041

Independent complex or single gene loci (r^2^<0.2) with SNP(s) with a conditional FDR (condFDR)<0.05 in schizophrenia (SCZ) given the association in bipolar disorder (BD). We defined the most significant SCZ SNP in each LD block based on the minimum condFDR for BD. The most significant SNPs in each LD block are listed. All loci with SNPs with condFDR<0.05 were used to define the number of the loci. Chromosome location (Chr). SCZ FDR values<0.05 are in bold.

† Same locus identified in previous SCZ genome-wide association studies. All data were first corrected for genomic inflation.

### Bipolar disorder gene loci identified with conditional FDR

**Figure 3:**
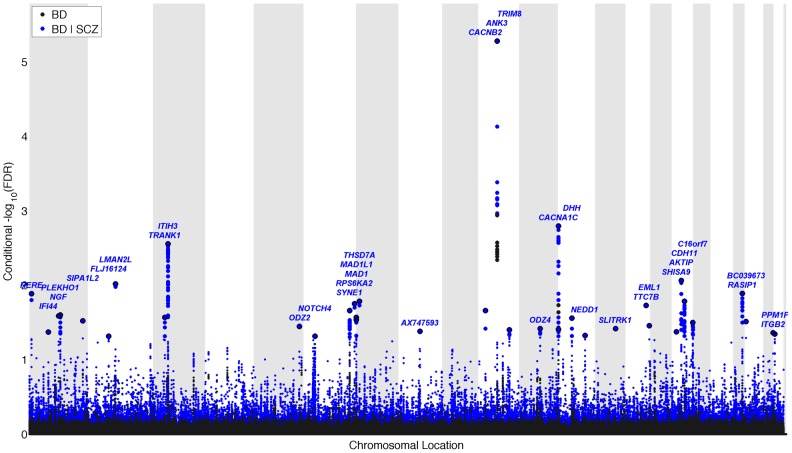
“Conditional Manhattan plot” of conditional −log_10_ (FDR) values for Bipolar disorder (BD) alone (black) and BD given schizophrenia (SCZ; BD|SCZ, blue). SNPs with conditional −log_10_ FDR>1.3 (i.e. FDR<0.05) are shown with large points. A black line around the large points indicates the most significant SNP in each LD block and this SNP was annotated with the closest gene, which is listed above the symbols in each locus. The figure shows the localization of significant loci. Details about the loci are provided in Table 2.

We constructed a “conditional” Manhattan plot for bipolar disorder showing the FDR conditional on schizophrenia (Figure 3) and identified significant loci on a total of 16 chromosomes (1–3, 5–8, 10–14, 16 and 19–22) associated with bipolar disorder leveraging the reduced FDR obtained by the associated schizophrenia phenotype. To estimate the number of independent loci, we pruned the associated SNPs (removed SNP with LD >0.2), and identified a total of 35 independent loci with a significance threshold of conditional FDR<0.05 (Table 2). Of these, one locus was complex, i.e. included several significant SNPs, and the rest were single gene loci. Using the more conservative conditional FDR threshold of 0.01, 5 independent loci remained significant. The most significant locus was close to *ANK3* on chromosome (10q21). This is the only locus that would have been discovered using standard methods based on p-values (Bonferroni correction). Using the FDR method in bipolar disorder alone, an additional locus was identified, close to *CACNA1C* (12p13.3). Both these loci have been discovered earlier [14], [31]. The remaining 33 loci would not have been identified in the current sample without using the pleiotropy-informed conditional FDR method. Of these, the regions close to *SYNE1* (6q25) and *ODZ4* (11q14.1) have been identified in earlier GWAS after including large replication samples [14], [32]. Of interest, the *ITIH3* region (3p21.1), *ANK3* (10q21) and *CACNA1C* (12p13.3) were discovered previously in the same, combined schizophrenia and bipolar disorder sample [13], [14]. Thus, pleiotropy-informed conditional FDR validated 5 loci discovered in considerably larger samples, and discovered 30 new loci.

**Table 2:** Conditional FDR; BD loci given SCZ (BD|SCZ).

locus	SNP	neighbor gene	Chr	pval BD	fdr BD	fdr BD|SCZ
1	rs2252865	*RERE*	*1p36.23*	2.19E-04	0.44657	0.01306
2	rs4650608	*IFI44*	*1p31.1*	1.00E-03	0.64629	0.04250
3	rs10776799	*NGF*	*1p13.1*	9.68E-06	0.17368	0.02579
4	rs7521783	*PLEKHO1*	*1q21.2*	5.58E-04	0.57626	0.02503
5	rs573140	*SIPA1L2*	*1q42.2*	6.58E-06	0.15946	0.03009
6	rs3911862	*FLJ16124*	*2p14*	5.65E-05	0.26909	0.04864
7	rs2271893	*LMAN2L*	*2q11.2*	1.85E-05	0.18928	0.00960
8	rs9834970	*TRANK1*	*3p22.2*	5.20E-04	0.57626	0.02711
9	rs2535629	*ITIH3* †	*3p21.1*	1.29E-05	0.17896	0.00279
10	rs2902101	*ODZ2*	*5q34*	1.04E-04	0.33589	0.03570
11	rs3134942	*NOTCH4*	*6p21.3*	1.15E-03	0.66028	0.04844
12	rs9371601	*SYNE1* †	*6q25*	1.10E-06	0.06351	0.02196
13	rs3823198	*RPS6KA2*	*6q27*	4.16E-05	0.22281	0.01779
14	rs4332037	*MAD1*	*7p22*	3.97E-05	0.22281	0.02918
15	rs6461233	*MAD1L1*	*7p22*	5.19E-04	0.57626	0.02711
16	rs10277665	*THSD7A*	*7p21.3*	5.42E-05	0.24328	0.01641
17	rs6982836	*AX747593*	*8q13.2*	5.64E-05	0.26909	0.04168
18	rs7083127	*CACNB2*	*10p12*	1.40E-04	0.37364	0.02191
19	rs10994359	*ANK3* †	*10q21*	**8.12E-10**	**0.00115**	0.00001
20	rs10883757	*TRIM8*	*10q24.3*	1.11E-03	0.64629	0.03991
21	rs17138230	*ODZ4* †	*11q14.1*	1.43E-05	0.18382	0.03822
22	rs2239037	*CACNA1C*	*12p13.3*	9.06E-04	0.64629	0.03928
	rs10774037	*CACNA1C* †	*12p13.3*	2.42E-07	**0.01859**	0.00161
23	rs7296288	*DHH*	*12q13.1*	2.88E-05	0.20749	0.02777
24	rs12427050	*NEDD1*	*12q23.1*	5.00E-04	0.57626	0.04728
25	rs4390476	*SLITRK1*	*13q31.1*	2.03E-04	0.44657	0.03843
26	rs961196	*TTC7B*	*14q32.11*	2.96E-04	0.50926	0.01872
27	rs11160562	*EML1*	*14q32*	6.93E-04	0.60769	0.03496
28	rs12708772	*SHISA9*	*16p13.12*	9.89E-04	0.64629	0.04219
29	rs11863156	*AKTIP*	*16q12.2*	7.86E-05	0.30029	0.00865
30	rs1424003	*CDH11*	*16q21*	5.54E-05	0.24328	0.01641
31	rs3809646	*C16orf7*	*16q24*	5.76E-04	0.60769	0.03171
32	rs281393	*RASIP1*	*19q13.33*	5.99E-05	0.26909	0.01293
33	rs159788	*BC039673*	*20p13*	6.48E-04	0.60769	0.03080
34	rs3746972	*ITGB2*	*21q22.3*	1.42E-04	0.41109	0.04369
35	rs381523	*PPM1F*	*22q11.22*	1.28E-03	0.66028	0.04536

For the independent complex or single gene loci (r^2^<0.2) with SNP(s) with a conditional FDR (condFDR)<0.05 in bipolar disorder (BD) given association with schizophrenia (SCZ). All independent loci are listed consecutively. Chromosome location (Chr). All data were first corrected for genomic inflation. BD FDR values<0.05 are in bold.

† Same locus identified in previous BD genome-wide association studies.

### Pleiotropic gene loci in both schizophrenia and bipolar disorder identified with conjunctional FDR

**Figure 4:**
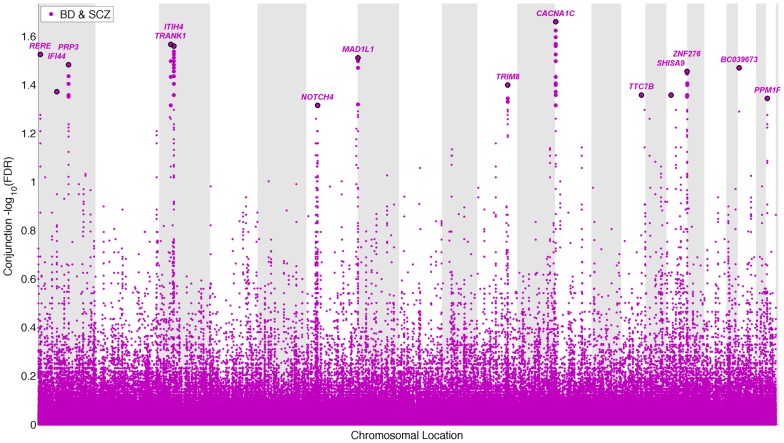
“Conjunction Manhattan plot” of conjunction −log_10_ (FDR) values for schizophrenia (SCZ) and bipolar disorder (BD). SNPs with −log_10_ (FDR)>1.3, (i.e. FDR<0.05) for both SCZ and BD are shown with large points. A black line around the large points indicates the most significant SNP in each LD block and this SNP was annotated with the closest gene, which is listed above the symbols in each chromosome. The figure shows the localization of the ‘pleiotropic loci’, and further details are provided in Table 3.

To identify pleiotropic loci in schizophrenia and bipolar disorder, we performed a conjunction FDR analysis, using this to construct a “conjunction” Manhattan plot (Figure 4). We detected 14 independent pleiotropic loci (pruned based on LD>0.2, black line around large circles) with conjunction FDR<0.05, all single gene loci, located on a total of 10 chromosomes (chr. 1, 3, 6, 7, 10, 12, 14, 16, 20, 22 – for further details, please see Table 3). Of these loci, 3 have been implicated in bipolar disorder and schizophrenia earlier: *NOTCH4* (6p21.2) with schizophrenia using a larger replication sample [13], [17], and the *ITIH4* (3p21.1), and *CACNA1C* (12p13.3) regions, both discovered previously in the same, combined schizophrenia and bipolar disorder sample [13], [14]. Interestingly only one conjunctional locus was found on chromosome 6, suggesting that there are several schizophrenia loci on this chromosome not overlapping with bipolar disorder. The *ANK3* locus was not indicated in the conjunctional analysis, which indicates that the overlap is mostly driven by the association in bipolar disorder (Table 2). The direction of the effect (z-scores) across all the pleiotropic SNPs was the same for bipolar disorder and schizophrenia, except for locus 33 (*BC039673*, 20p13), which could be due to differences in LD structure in this region. These findings suggest overlapping genetic pathways in schizophrenia and bipolar disorders.

**Table 3:** Conjunction FDR; pleiotropic loci in SCZ and BD (SCZ&BD).

locus	SNP	neighbor gene	Chr	A1	A2	conjfdr BD&SCZ	z-score BD	z-score SCZ
1	rs2252865	*RERE*	*1p36.23*	T	C	0.030	3.696	3.494
2	rs4650608	*IFI44*	*1p31.1*	T	C	0.043	3.289	3.711
4	rs11205362	*PRP3*	*1q21.1*	G	A	0.033	3.404	3.262
8	rs9834970	*TRANK1*	*3p22.2*	C	T	0.027	3.470	3.965
9	rs4687657	*ITIH4* †	*3p21.1*	G	T	0.028	3.787	3.781
11	rs3134942	*NOTCH4* †	*6p21.3*	G	T	0.048	3.251	3.571
15	rs3757440	*MAD1L1*	*7p22*	A	G	0.031	3.490	3.425
20	rs10883757	*TRIM8*	*10q24.3*	C	T	0.040	3.261	3.046
22	rs1006737	*CACNA1C* †	*12p13.3*	A	G	0.022	4.553	4.137
26	rs961196	*TTC7B*	*14q32.11*	C	T	0.044	3.618	2.960
28	rs12708772	*SHISA9*	*16p13.12*	C	T	0.044	3.294	2.955
31	rs1800359	*ZNF276*	*16q24.3*	A	G	0.035	3.329	3.165
33	rs159788	*BC039673*	*20p13*	G	A	0.034	3.411	−3.232
35	rs381523	*PPM1F*	*22q11.22*	A	G	0.045	3.220	3.166

Independent complex or single gene loci (r^2^<0.2) with SNP(s) with a conjunctional FDR (conjFDR)<0.05 in schizophrenia (SCZ) *and* bipolar disorder (BD). All SNPs with a conjFDR value<0.05 (bidirectional association, i.e. association with SCZ given association with BD (condFDR<0.05) and association with BD given association with SCZ (condFDR<0.05)) are listed and sorted in each LD block. We defined the most significant SNP in each LD block based on the minimum conjFDR. All independent loci are listed consecutively, and the same locus number are used as in the condFDR<0.05 results (Table 1). Chromosome (Chr). Z-scores for each pleiotropic locus are provided, with minor allele (A1) and major allele (A2). All data were first corrected for genomic inflation.

† Same locus identified in previous BD or SCZ genome-wide association studies.

### Model-based power analyses

**Figure 5:**
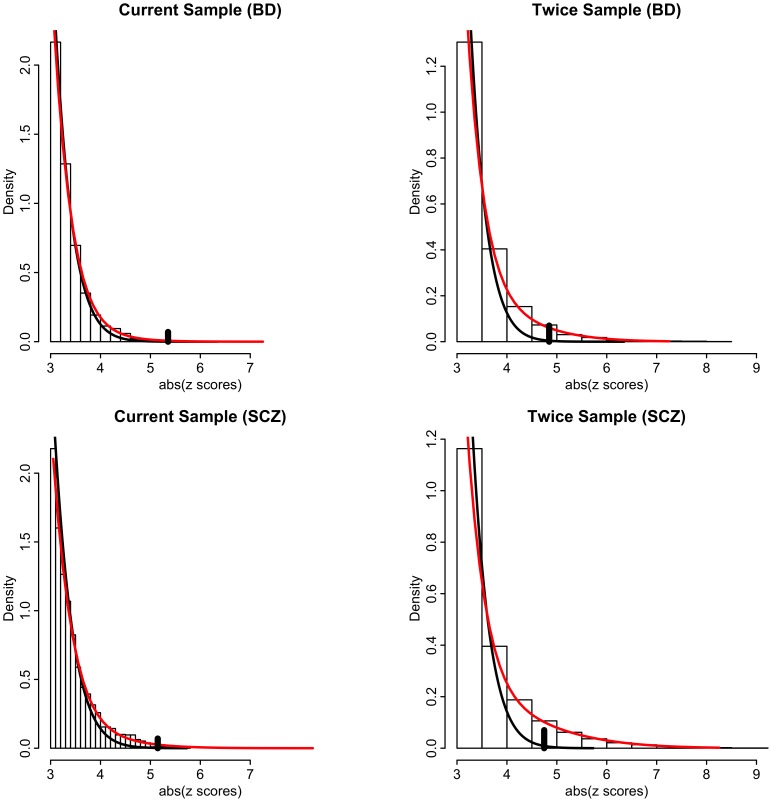
Histograms of absolute z-scores for bipolar disorder (BD, top panels) and schizophrenia (SCZ, bottom panels) for z-scores ≥3. Left panels are actual data, whereas right panels are hypothetical realizations from a doubling of effective sample size, generated from mixture model fits of f(z) = π_0_f_0_(z)+(1−π_0_)f_1_(z) (see Text S1). Black lines are null sub-densities π_0_f_0_(z) and red lines are the full mixture densities f(z). The local false discovery rate is the ratio fdr = π_0_f_0_(z)/f(z). Vertical black bars in each plot indicate the cut-points where local fdr≤0.05.

Our model-free conditional FDR analyses circumvent the issue of bias due to model misspecification. However, to ascertain the impact of effective sample size and conditioning on relative power over using unconditioned FDR on current sample sizes, it is necessary to use a model-based approach that estimates the proportion and distribution of non-null SNPs [33]. We thus posit a mixture of null and non-null Gaussian distributions [34] (see Methods and Text S1). Resulting model fits are displayed in Figure 5 for schizophrenia and bipolar disorder for absolute z scores ≥3. Left panels are actual data, whereas right panels are hypothetical realizations from a doubling of effective sample size, generated from mixture model fits. Null densities largely coincide with the overall densities except for z scores with absolute value larger than 4, at which point the ratio of null to total SNPs, equal to the *local false discovery rate* (local FDR), is less than 0.5 (left panels of Figure 5). Thus, while highly polygenic, most non-null SNPs have local FDR much larger than 0.05. The local FDR does not drop below 0.05 until absolute z scores exceed 5. Far more of the “hidden” non-null SNPs lie below this significance threshold than above it. Many of these hidden SNPs lie just below the significance threshold, so that an effective doubling of the sample size produces a ∼30 times increase in number of rejected non-null SNPs with local FDR ≤0.05 (right panels of Figure 5).

**Figure 6:**
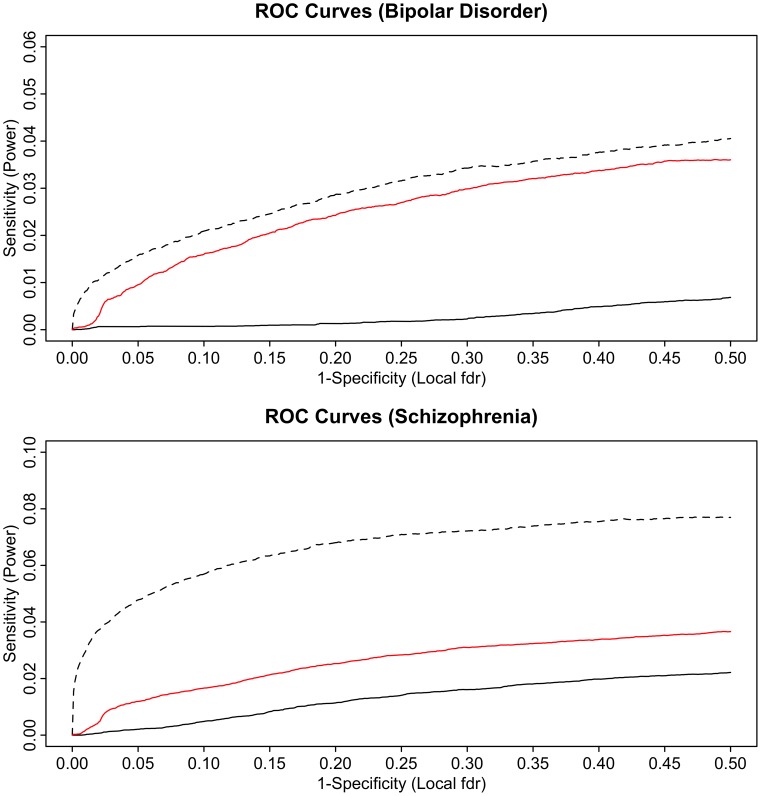
ROC curves for bipolar disorder (top) and schizophrenia (bottom). Solid black line show the proportion of non-null SNPs declared significant (sensitivity) for a given local false discovery rate (1-specificity). The corresponding ROC curves for bipolar disorder local FDR conditional on schizophrenia (top) and schizophrenia local FDR conditional on bipolar disorder (bottom) are given in red. Power resulting from a hypothetical doubling of effective subject sample size is given by the dashed black lines.

Another model-based analysis using a bivariate mixture of Gaussians showed that a very high proportion of the non-null schizophrenia SNPs are also non-null for bipolar disorder (and *vice versa*) leading to large increases in power when using the conditional FDR approach. This increase in power is also due to the large number of SNPs with p-values just below the Bonferroni threshold. Figure 6 shows the power, or sensitivity to detect non-null SNPs for differing local FDR cut points from unconditional and conditional local FDR and, for comparison, from a hypothetical doubling of the number of subjects. Using conditional over unconditional local FDR results in an increase of 15–20 times the number of non-null SNPs discovered for a local FDR≤0.05. The increase in power for conditional FDR, while dramatic, is not as large as what would be obtained by doubling the sample size. This is not unexpected, given that the highly polygenic non-null SNPs for schizophrenia and bipolar disorder, many just below the given significance thresholds, are largely but not completely overlapping. Note, given their highly polygenic distribution the vast majority of non-null SNPs remain undiscovered even using conditional FDR approaches or under an effective doubling of the number of subjects.

To test for enrichment with a “control trait” with little or no polygenic overlap with psychiatric disease, we performed pleiotropy analysis using type 2 diabetes (T2D) GWAS data. The analyses confirmed that there was a very small level of pleiotropic enrichment between schizophrenia and T2D, leading to little if any improvement in statistical power (See Text S1 and Figure S7).

## Discussion

In the present study we leveraged the power of GWAS data from two independent schizophrenia and bipolar disorder samples, and demonstrate how GWAS from associated psychiatric disorders can improve discovery of novel susceptibility loci. Using standard GWAS analytical methods, we identified only one significant locus. By applying traditional FDR methods in the separate GWAS samples, we found an additional 6 loci (2 in bipolar disorder, 4 in schizophrenia). Combining the independent schizophrenia and bipolar disorder GWAS samples, we identified a total of 58 loci in schizophrenia and 35 in bipolar disorders, with conditional FDR<0.05 as a threshold. Nine of the current loci have been identified earlier in larger samples using standard GWAS analytical methods (7 in schizophrenia, 5 in bipolar disorder, and 3 in combined samples), while 10 other loci have been reported to show borderline association with bipolar disorder or schizophrenia (Table S1). These results demonstrate the feasibility of using a cost-effective, pleiotropy-informed conditional FDR approach to discover common variants in schizophrenia and bipolar disorders.

The proposed statistical approach is based on the observation that all SNPs should not be treated as exchangeable. Rather, a SNP with large effects in two associated phenotypes has a higher probability of being a true non-null effect, and hence also a higher probability of being replicated in independent studies. We thus applied a conditional FDR approach we have previously developed for GWAS p-values [36], adapted from methods originally used for linkage analysis and microarray expression data [5], [37]. Decreased conditional FDR (equivalently, increased conditional TDR) for a given nominal p-value increases power to detect true non-null effects. Increased conditional TDR is directly related to increased replication effect sizes and replication rates in *de novo* samples. Using this conditional approach we were able to increase power to detect true non-null signals in independent studies for given nominal p-values cut-offs. Equivalently, in the conditional approach the FDR can be used to control FDR at a given level while increasing power to discover non-null SNPs over approaches that treat all SNPs as interchangeable. We also applied a previously developed conjunction FDR approach [36] to investigate which SNPs are pleiotropic, impacting risk of both schizophrenia and bipolar disorder. The conjunction statistic used is the maximum of the conditional FDR for schizophrenia given bipolar disorder and *vice versa*. SNPs that exceed a stringent conjunction threshold are thus highly likely to be non-null in the two phenotypes simultaneously.

The extra number of significant loci identified in the current study compared to ‘conventional’ GWAS methods is remarkable. The power analyses suggest that the large increase in power is due to the conditional FDR method, and not an implicit higher false discovery rate. Compared to conventional GWAS methods, traditional FDR methods only identified a few extra loci. The large increase in power came from using conditional FDR, which identified 14.5 times as many schizophrenia SNPs and 17.5 times as many bipolar SNPs (at FDR≤.05 level) compared to traditional FDR methods. This large increase in power seems to be due to two factors: the highly polygenic distribution of non-null SNPs and the high degree of pleiotropy between schizophrenia and bipolar disorder. We quantified this using a model-based mixture of null and non-null Gaussian distributions [34]. Mixture models estimate roughly 1.2% of tag SNPs are non-null in both bipolar disorder and schizophrenia. With over 1 million assayed SNPs in common between both phenotypes, the number of un-pruned, non-null SNPs is thus in excess of 12,000 in each phenotype. The vast majority of these non-null SNPs are hidden within the large proportion (∼99%) of null SNPs. Results are in line with recent findings of a high proportion of variation in schizophrenia susceptibility captured by common SNPs [6]. Taken together, these findings strongly suggest that Empirical Bayes methods, as outlined by Efron [27] should be the method of choice for analyzing GWAS of polygenic human phenotypes, and for leveraging pleiotropy with other complex humans traits.

The current findings of polygenic enrichment suggest that genetic pleiotropy is important in severe mental disorders, as has been indicated earlier [13]–[15], [24], [25]. However, by using conditional FDR, we were able to leverage the overlapping polygenetic architecture to identify more of the specific SNPs involved. The current approach identified 58 loci in schizophrenia compared to 7 in the original publication [13]. In bipolar disorder, the added power from schizophrenia GWAS identified 35 loci compared to two loci in the original study [14]. It is important to note that this improvement in gene discovery was obtained despite the much smaller number of controls in the current analyses because the original analyses of the two disorders used largely overlapping control samples. Since we used data from the 1000 Genomes Project (1KGP) to calculate LD structure, the number of loci can vary somewhat compared to the original analysis. For both disorders, most of the current findings were borderline significant in the original GWAS mega-analysis, or identified in other GWAS of partly overlapping samples, such as *TRANK1* [38] and *SYNE1* [32]. Several of the currently identified genes have been associated in previous candidate gene studies, such as *DAOA* [39].

Further, we identified 14 loci strongly associated with *both* disorders, compared to three in the original combined analysis [13], [14]. Previous studies have mainly used Fisher combined tests for joint analysis, which test the null-hypothesis of no association in *any* phenotype, which means that the signal can be driven by one of the phenotypes. In contrast, conjunction FDR analyses assess the evidence that *either* phenotype is non-null. It is therefore difficult to directly compare the current findings with previous results. However, of the three identified loci in previous combined analysis [13], [14], both the *ITIH3-4* and *CACNA1C* regions were confirmed with the conjunctional analyses, but not the *ANK3* region. We found the latter to be associated with bipolar disorder in the current analysis, which suggests that previous results found with Fisher combined statistics were driven by the stronger association in bipolar disorder [13], [14].

The current findings suggest some interesting gene candidates related to overlapping biology of bipolar disorder and schizophrenia. The Major Histocompatibility Complex loci associations with schizophrenia in previous studies [13], [17] seem not to be strengthened by the combined analysis with bipolar disorder, as they are minimally represented among the current pleiotropic loci (conjunction FDR analyses). The only pleiotropic gene on chromosome 6 was *NOTCH 4*, which has recently also been implicated in bipolar disorder [26], . The current findings strengthen the involvement of genes related to calcium homeostasis and receptor functioning. In schizophrenia, both *CACNA1C* and *ANK3* were identified, and in bipolar disorder *TRANK1* and *CACNB2* were also significantly associated. *CACNA1C* and *CACNB2* are related to key proteins involved in unifying the generation of calcium spikes in neocortical pyramidal neurons, which is a closely integrated process [41]. It is likely that such functional processes could be involved in generation of symptoms in severe mental disorders, and may thus be a potential therapeutic target. Interestingly, *PPM1F*, a Mg2+/Mn2+ dependent protein phosphatase, related to calcium/calmodulin-dependent protein kinase II gamma, was also associated with both disorders, and seems to further strengthen the hypothesis that alterations in electrophysiological function play a role in the pathophysiology of these disorders. It is also noteworthy that SNPs located close to *MAD1L1* were significantly associated with both schizophrenia and bipolar disorder. *MAD1L1* is located in a human accelerated region in the genome, which shows a large difference between humans and chimpanzees [42], and thus is suggested to be involved in human-specific traits.

In addition to uncovering more of the missing heritability of bipolar disorder and schizophrenia, the current findings support the notion that genetic pleiotropy is important for variation in human phenotypes [9], and suggest that there is substantial polygenic pleiotropy between bipolar disorder and schizophrenia which warrants further exploration. In the current study we defined pleiotropy as a single gene or variant being associated with more than one distinct phenotype (diseases) [9]. It is possible that some of the loci identified in the current study are not pleiotropic but rather underlie common aspects of the schizophrenia and bipolar disorder phenotypes [9]. This possibility warrants further investigation, but requires samples with more detailed information on clinical characteristics. In the current analyses we focused on SNPs, but gene-based pleiotropy is also of interest [10], as is the use of the current approach for developing methods for risk prediction across traits. However, these applications require raw data from individual participants and these data are not currently available.

In conclusion, the current findings demonstrate that in schizophrenia and bipolar disorder, pleiotropy-informed conditional FDR can improve the statistical power for detecting novel polygenic effects. Results from conditional and conjunction FDR analyses also offer insights into potential shared mechanistic relationships between these two mental disorders.

## Materials and Methods

### Ethics statement

The relevant institutional review boards or ethics committees approved the research protocol of the individual GWAS used in the current analysis and all human participants gave written informed consent.

### Participant samples

We obtained complete GWAS results in the form of summary statistics p-values from the Psychiatric GWAS Consortium (PGC) – Schizophrenia and Bipolar Disorder Working Groups. The schizophrenia (SCZ) GWAS summary statistics results were obtained from the PGC Schizophrenia Work Group [13], which consisted of 9,394 cases with schizophrenia or schizoaffective disorder and 12,462 controls (52% screened) from a total of 17 samples from 11 countries. Semi-structured interviews were used by trained interviewers to collect clinical information, and operational criteria were used to establish diagnosis. The quality of phenotypic data was verified by a systematic review of data collection methods and procedures at each site, and only studies that fulfilled these criteria were included. Controls were selected from the same geographical and ethnic populations as cases. For further details on sample characteristics and quality control procedures applied, please see Ripke et al..

The bipolar disorder (BD) GWAS summary statistics results were obtained from the PGC Bipolar Disorder Working Group [14], which consisted of n = 16,731 participants, including 7481 cases and 9250 controls, from 11 studies from 7 countries. Standardized semi-structured interviews were used by trained interviewers to collect clinical information about lifetime history of psychiatric illness and operational criteria applied to make lifetime diagnosis according to recognized classifications. All cases have experienced pathologically relevant episodes of elevated mood (mania or hypomania) and meet operational criteria for a BD diagnosis. The sample consisted of BD I (84%), BD II (11%), schizoaffective disorder bipolar type (4%), and BD NOS (1%). Controls were selected from the same geographical and ethnic populations as cases. For further details on sample characteristics and quality control procedures applied, please see Sklar et al. [14].

Due to overlapping control samples in these studies, the common controls were split randomly, and divided between the two case-control analyses. All results presented here are based on these non-overlapping control samples, with n = 9379 cases and n = 7736 control samples in schizophrenia, and n = 6990 cases and n = 4820 controls in bipolar disorder analyses.

### Statistical analyses

Analyses implemented here were motivated by previously published stratified FDR methods [5], [37]. However, we found that stratified empirical cdfs exhibited a high degree of variability. Instead, we computed empirical cdfs for the first phenotype conditional on nominal p-values of the second being at or below a given threshold. These conditional empirical cdfs vary more smoothly as a function of p-value thresholds in the second (associated) phenotype than do empirical cdfs employing disjoint strata. Conditional FDR estimates derived from the conditional empirical cdfs are a simple extension of Efron's Empirical Bayes FDR methods [33].

One advantage of the model-free empirical cdf approach is the avoidance of bias in conditional FDR estimates from model misspecification. However, there are inherent limitations to model-free approaches, especially with respect to inferring properties of the non-null distribution and, consequently, estimating power to detect non-null effects. We present complementary model-based analyses in the Supporting Information that estimate conditional and conjunctional local false discovery rate (fdr) [27]. Results presented in the Supporting Information using this model-based fdr corroborate the results of the model-free approaches presented here.

### Genomic control

The empirical null distribution in GWAS is affected by global variance inflation due to population stratification and cryptic relatedness [43] and deflation due to over-correction of test statistics for polygenic traits by standard genomic control methods [34]. We applied a control method leveraging only intergenic SNPs which are likely depleted for true associations (Schork et al., *under review*). First, we annotated the SNPs to genic (5′UTR, exon, intron, 3′UTR) and intergenic regions using information from the 1KGP. As illustrated in Figure S1, there is an enrichment of functional genic regions in schizophrenia compared to the intergenic SNP category. We used intergenic SNPs because their relative depletion of associations suggests that they provide a robust estimate of true null effects and thus seem a better category for genomic control than all SNPs. We converted all p-values to z-scores and for each phenotype we estimated the genomic inflation factor λ_GC_ for intergenic SNPs. We computed the inflation factor, λ_GC_ as the median z-score squared divided by the expected median of a chi-square distribution with one degree of freedom and divided all test statistics by λ_GC_. The conditional Q-Q plots for schizophrenia after control for genomic inflation are shown in Figure S1.

### Conditional Q–Q plots for assessing pleiotropic enrichment

To assess pleiotropic enrichment, we used Q-Q plots conditioned on ‘pleiotropic’ effects. For a given associated phenotype, enrichment for pleiotropic signals is present if the degree of deflection from the expected null line is dependent on SNP associations with the second phenotype. We constructed conditional Q-Q plots of empirical quantiles of nominal −log_10_(p) values for SNP association with schizophrenia for all SNPs, and for subsets of SNPs determined by the nominal p-values of their association with bipolar disorder being at or below a given threshold. Specifically, we computed the empirical cumulative distribution of nominal p-values for a given phenotype for all SNPs and for SNPs with significance levels below the indicated cut-offs for the other phenotype (−log_10_(p)≥0, −log_10_(p)≥1, −log_10_(p)≥2, −log_10_(p)≥3 corresponding to p≤1, p≤0.1, p≤0.01, p≤0.001, respectively). The nominal p-values (−log_10_(p)) are plotted on the y-axis, and the empirical quantiles (−log_10_(q), where q = 1- empirical cdf(p)) are plotted on the x-axis. To assess for polygenic effects below the standard GWAS significance threshold, we focused the conditional Q-Q plots on SNPs with nominal −log_10_(p)<7.3 (corresponding to p>5×10^−8^).

### Conditional false discovery rate

Enrichment seen in the conditional Q-Q plots can be directly interpreted in terms of the FDR. Specifically, for a given p-value cutoff, the Bayes FDR [33], closely related to the q-value of Storey [44] is defined as
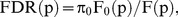
(1)where π_0_ is the proportion of null SNPs, F_0_ is the null cdf, and F is the cdf of all SNPs, both null and non-null; see Text S1 for details on this simple mixture model formulation [33]. Under the null hypothesis, F_0_ is the cdf of the uniform distribution on the unit interval [0,1], so that Eq. [1] reduces to
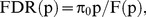
(2)The cdf F can be estimated by the empirical cdf q = Ν_p_/Ν, where Ν_p_ is the number of SNPs with p-values less than or equal to p, and N is the total number of SNPs. Replacing F by q and replacing π_0_ with unity in Eq. [2], we get
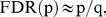
(3)which is biased upwards as an estimate of Eq. [2] [33]. If π_0_ is close to one, as is likely true for most GWAS, the increase in bias by setting it to unity in Eq. [3] is minimal. The quantity 1 – p/q, is therefore biased downward, and hence is a conservative estimate of the TDR = 1 - FDR. Note, Eq. [3] is the Empirical Bayes estimate of the Bayesian FDR described by Efron [33]. Referring to the formulation of the Q-Q plots, we see that Eq. [3] is equivalent to the nominal p-value divided by the empirical quantile, as defined earlier. Given the −log_10_ construction of the Q-Q plots we easily obtain
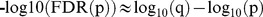
(4)demonstrating that the (conservatively) estimated FDR is directly related to the horizontal shift of the curves in the conditional Q-Q plots from the expected line x = y, with a larger shift corresponding to a smaller FDR. This is illustrated in Figure 1. For each p-value threshold in the associated trait (e.g. bipolar disorder), we calculated the conditional TDR as a function of p-value in the primary trait (e.g. schizophrenia, indicated by different colored curves) in Figure 1 according to Eq. [4].

### Conditional statistics—probability of association with one disorder

We define the *conditional FDR* as the posterior probability that a given SNP is null for the first phenotype given that the p-values for both phenotypes are as small or smaller as the observed p-values. Formally, this is given by
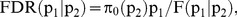
(5)where p_1_ is the p-value for the first phenotype, p_2_ is the p-value for the second, and F(p_1_ | p_2_) is the conditional cdf and π_0_(p_2_) the conditional proportion of null SNPs for the first phenotype given that p-values for the second phenotype are p_2_ or smaller. Eq. [5] makes the assumption, reasonable for independent GWAS, that summary statistics are independent across phenotypes if they are null for at least one phenotype. We produce a conservative estimate of FDR(p_1_ | p_2_) by setting π_0_(p_2_) = 1 and using the empirical conditional cdf in place of F(p_1_ | p_2_) in Eq. [5]. This is a straightforward generalization of the Empirical Bayes approach developed by Efron [33]. We assign a conditional FDR value for schizophrenia given bipolar disorder p-values (denoted by FDR _SCZ | BD_) to each SNP by computing conditional FDR estimates on a grid and interpolating these estimates into a two-dimensional look-up table (Figure S2). All SNPs with conditional FDR<0.05 (−log_10_(FDR)>1.3) in schizophrenia given association with bipolar disorder are listed in Table 1 after ‘pruning’ (removing all SNPs with r^2^>0.2 based on 1KGP LD structure). We used the same procedure, in the opposite direction, to assign a conditional FDR value (denoted as FDR _BD|SCZ_) for bipolar disorder given schizophrenia p-values to each SNP. All SNPs with FDR<0.05 (−log_10_(FDR)>1.3) in bipolar disorder given schizophrenia are listed in Table 2 after pruning. A significance threshold of FDR<0.05 nominally corresponds to 5 false positives per 100 reported associations. We present a complementary model-based approach to estimating conditional FDR in the Text S1.

### Conjunction statistics—test of association with both phenotypes

In order to identify which of the SNPs were associated with schizophrenia *and* bipolar disorder we used a conjunction FDR procedure similar to that described for p-value statistics in Nichols et al. [45]. This minimizes the effect of a single phenotype driving the common association signal. *Conjunction FDR* is defined as the posterior probability that a given SNP is null for both phenotypes simultaneously when the p-values for both phenotypes are as small or smaller than the observed p-values. Formally, conjunction FDR is given by
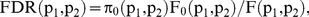
(6)where π_0_(p_1_, p_2_) is the proportion of SNPs null for both phenotypes simultaneously, F_0_(p_1_, p_2_) = p_1_ p_2_ is the joint null cdf, and F(p_1_, p_2_) is the joint overall cdf.

Conditional empirical cdfs provide a model-free method to obtain conservative estimates of Eq. [6]. This can be seen as follows. Estimate the conjunction FDR by
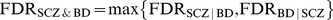
(7)where FDR_ SCZ | BD_ and FDR_ BD | SCZ_ (the estimated conditional FDRs described above) are conservative (upwardly biased) estimates of Eq. [5]. Thus, Eq. [7] is a conservative estimate of max{p_1_/F(p_1_ | p_2_), p_2_/F(p_2_ | p_1_)} = max{p_1_ F_2_(p_2_)/F(p_1_, p_2_), p_2_ F_1_(p_1_)/F(p_1_, p_2_)}. For enriched samples, p-values will tend to be smaller than predicted from the uniform distribution, so that F_1_(p_1_)≥p_1_ and F_2_(p_2_)≥p_2_. Hence, max{p_1_ F_2_(p_2_)/F(p_1_, p_2_), p_2_ F_2_(p_1_)/F(p_1_, p_2_)}≥max{p_1_ p_2_/F(p_1_, p_2_), p_2_ p_1_/F(p_1_, p_2_)} = p_1_ p_2_/F(p_1_, p_2_)≥π_0_(p_1_, p_2_) p_1_ p_2_/F(p_1_, p_2_). The last quantity is precisely the conjunction FDR defined by Eq. [6]. Thus, Eq. [7] is a conservative model-free estimate of the conjunction FDR. We present a complementary model-based approach to estimating conjunction FDR in the Text S1.

We assigned the conjunction FDR values by interpolation into a bi-directional two-dimensional look-up table (Figure S3). All SNPs with conjunction FDR<0.05 (−log_10_(FDR)>1.3) with schizophrenia and bipolar disorder considered jointly are listed in Table 3 (after pruning), together with the corresponding z-scores and minor alleles. The z-scores were calculated from the p-values and the direction of effect was determined by the risk allele.

### Conditional Manhattan plots

To illustrate the localization of the genetic markers associated with schizophrenia given their association with bipolar disorder, and *vice versa*, we used a ‘Conditional Manhattan plot’, plotting all SNPs within an LD block in relation to their chromosomal location. As illustrated in Figure 2 for schizophrenia, the large points represent the SNPs with conditional FDR<0.05, whereas the small points represent the non-significant SNPs. All SNPs without ‘pruning’ (removing all SNPs with r^2^>0.2 based on 1KGP LD structure) are shown. The strongest signal in each LD block is illustrated with a black line around the circles. This was identified by ranking all SNPs in increasing order, based on the conditional FDR value for schizophrenia given bipolar disorder, and then removing SNPs in LD r^2^>0.2 with any higher ranked SNP. Thus, the selected locus was the most significantly associated with schizophrenia in each LD block (Figure 2). A similar procedure was used in the conditional Manhattan plot for bipolar disorder given schizophrenia (Figure 3).

### Conjunction Manhattan plots

To illustrate the localization of the pleiotropic genetic markers associated with both schizophrenia and bipolar disorder, we present a ‘Conjunction Manhattan plot’, plotting all SNPs with a significant conjunction FDR within an LD block in relation to their chromosomal location. As illustrated in Figure 4, the large points represent the significant SNPs (FDR<0.05), whereas the small points represent the non-significant SNPs. All SNPs without ‘pruning’ (removing all SNPs with r^2^>0.2 based on 1KGP LD structure) are shown, and the strongest signal in each LD block is illustrated with a black line around the circles. We ranked all SNPs based on the conjunction statistic and removed SNPs in LD r^2^>0.2 with any higher ranked SNP.

### Model-based power analyses

While model-free approaches avoid assumptions that may bias results, it is necessary to take a model-based approach for assessing the power to detect non-null SNPs [33]. As in Eq. [1], let π_0_ be the proportion of null SNPs and let π_1_ = 1−π_0_ be the proportion of non-null SNPs. Following Yang et al. [34], the probability density f(z_i_) of the test statistic (z score) for the *i*th SNP is given by
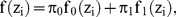
(8)where the null density f_0_(z_i_) corresponds to a N(0, σ_0_^2^) distribution and the non-null density f_1_(z_i_) corresponds to a N(0, σ_0_^2^+σ_1_^2^) distribution. Both σ_0_^2^ and σ_1_^2^ are estimated from the data (see Text S1). The *lo*c*al false discovery rate*, defined as the posterior probability that a SNP is non-null given the observed z score, is given by Efron and Tibshirani [35]

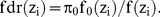
(9)Using this mixture of Gaussians formulation, we can assess relative power for gene discovery by determining the proportion of non-null SNPs with local fdr less than a given cut-off, e.g., 0.05. We can also determine the impact of scaling the effective sample size on the distribution f_1_(z_i_) of non-null SNPs.

We extend this model to a bivariate framework by postulating a four groups model of bivariate Gaussians. Let **z**_i_ be the bivariate z scores for the *i*th SNP for schizophrenia and bipolar disorder. The four groups mixture model is given by
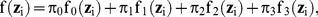
(10)where π_0_ is the proportion of SNPs which are null for both phenotypes, π_1_ and π_2_ are the proportion of SNPs which are non-null for schizophrenia and null for bipolar disorder (and *vice versa*), and π_3_ is the proportion of SNPs non-null for both simultaneously. The component densities f_0_, f_1_, f_2_, and f_3_ are bivariate Gaussian with zero mean and variance-covariance matrices estimated from the data. From model [9], we can compute conditional local fdr, similar to the conditional FDR described above. We can also determine the degree of pleiotropy from the estimated value of π_3_. Details of the methods for mixture models, local false discovery rate, and estimates of polygenicity, the degree of pleiotropic overlap, and power are presented in Text S1 and Figures S4, S5, S6, S7.

## Supporting Information

Figure S1 Stratified Q-Q plots of nominal versus empirical -log_10_ p-values of genic vs. intergenic regions, controlling for genomic inflation in schizophrenia (p<5×10^−8^). The plots illustrate the enrichment of genic SNPs compared to all SNPs, and lack of enrichment for intergenic SNPs. The plot in *top panel* is based on uncorrected data, showing inflation (λ 1.24). The intergenic SNPs were used for genomic control. The plot in *bottom panel* is based on data after correcting for inflation.
(DOC)

Figure S2 Based on the combination of p-value for the SNP in schizophrenia (SCZ) and bipolar disorder (BD), we assigned a conditional FDR value for SCZ to each SNP, by interpolation into a 2-dimensional lookup table. This is shown in *upper panel* for SCZ conditioned on BD, denoted FDR_SCZ | BD_. BD conditioned on SCZ, denoted FDR_BD|SCZ_ is shown in *lower panel*. Color scale refers to FDR values.
(DOC)

Figure S3 Based on the combination of p-value for the SNP in schizophrenia (SCZ) given bipolar disorder (BD), and the combination of p-value for the SNP in BD given SCZ, we assigned a conjunction FDR value for SCZ *and* BD to each SNP, by interpolation into a bi-directional 2-D look up table. This is denoted FDR_BD&SCZ_. Color scale refers to FDR values.
(DOC)

Figure S4 QQ-plot for schizophrenia (SCZ). Dotted lines give marginal fit based on MCMC estimates for the two-groups mixture model. Dashed line gives null distribution.
(DOC)

Figure S5 QQ-plot for bipolar disorder (BD). Dotted lines give marginal fit based on MCMC estimates for the two-groups mixture model. Dashed line gives null distribution.
(DOC)

Figure S6 QQ-plot for type 2 diabetes (T2D). Dotted lines give marginal fit based on MCMC estimates for the two-groups mixture model. Dashed line gives null distribution.
(DOC)

Figure S7 ROC curves for schizophrenia. Solid black line shows the proportion of non-null SNPs declared significant (Power; Sensitivity) for a given local false discovery rate (Local fdr; 1-specificity). The corresponding ROC curve for schizophrenia (SCZ) local false discovery rate conditional on type 2 diabetes (T2D) is given in red (SCZ | T2D). Red and black lines are nearly overlapping due to almost no increase in power. Power resulting from a hypothetical doubling of effective subject sample size is given by the dashed black line.
(DOC)

Table S1 Associated loci and previous GWAS findings.
(DOC)

Text S1 Supporting statistical methods.
(DOC)
