# Correction: Improved Detection of Common Variants Associated with Schizophrenia and Bipolar Disorder Using Pleiotropy-Informed Conditional False Discovery Rate

**DOI:** 10.1371/journal.pgen.1005544

**Published:** 2015-11-05

**Authors:** Ole A. Andreassen, Wesley K. Thompson, Andrew J. Schork, Stephan Ripke, Morten Mattingsdal, John R. Kelsoe, Kenneth S. Kendler, Michael C. O'Donovan, Dan Rujescu, Thomas Werge, Pamela Sklar, The Psychiatric Genomics Consortium (PGC), Bipolar Disorder and Schizophrenia Working Groups, J. Cooper Roddey, Chi-Hua Chen, Linda McEvoy, Rahul S. Desikan, Srdjan Djurovic, Anders M. Dale

**Incorrect mathematical definition of the conjunction FDR in the section ‘Conjunction statistics—test of association with both phenotypes’**

Under the sub-heading of ‘Conjunction statistics—test of association with both phenotypes’ in the ‘Materials and Methods’ section of the manuscript, there are errors in the mathematical definition of the conjunction FDR. The authors have provided an updated version here with corrections to the text in bold:

In order to identify which of the SNPs were associated with schizophrenia and bipolar disorder we used a conjunction FDR procedure similar to that described for p-value statistics in Nichols et al. [45]. This minimizes the effect of a single phenotype driving the common association signal. Conjunction FDR is defined as the posterior probability that a given SNP is null **for either phenotype or** both phenotypes simultaneously when the p-values for both phenotypes are as small or smaller than the observed p-values. Formally, conjunction FDR is given by

$${\mathrm{FDR}}_{\mathrm{SCZ}\&\mathrm{BD}}\left(p_{1},\;p_{2}\right)=\pi_{0}\;F_{0}\left(p_{1,}\;p_{2}\right)/\;F\left(p_{1,}\;p_{2}\right)+\pi_{1}\;F_{1}\left(p_{1,}\;p_{2}\right)/\;F\left(p_{1,}\;p_{2}\right)+\pi_{2}\;F_{2}\left(p_{1,}\;p_{2}\right)/\;F\left(p_{1,}\;p_{2}\right),$$ (6)

**where π_0_ is the *a priori* proportion of SNPs null for both SCZ and BD simultaneously and F_0_(p_1,_ p_2_) is the joint null cdf, π_1_ is the *a priori* proportion of SNPs non-null for SCZ and null for BD with F_1_(p_1,_ p_2_) the joint cdf of these SNPs, and π_2_ is the *a priori* proportion of SNPs non-null for BD and null for SCZ, with joint cdf F_2_(p_1,_ p_2_). F(p_1,_ p_2_) is the joint overall mixture cdf for all SCZ and BD SNPs.**

Conditional empirical cdfs provide a model-free method to obtain conservative estimates of Eq (6). This can be seen as follows. Estimate the conjunction FDR by

$${\text{FDR}}_{\text{SCZ}\&\text{BD}}=\max\{{\text{FDR}}_{\text{SCZ}|\text{BD}},{\text{FDR}}_{\text{BD}|\;\text{SCZ}}\}$$ (7)

where FDR_SCZ|BD_ and FDR_BD| SCZ_ (the estimated conditional FDRs described above) are conservative (upwardly biased) estimates of Eq. [5]. Thus, Eq (7) is a conservative estimate of max{p_1_/F(p_1_| p_2_), p_2_/F(p_2_|p_1_)} = **max{p**_**1**_ **F**_**2**_ **(p**_**2**_ **)/F(p**_**1**_ **, p**_**2**_ **), p**_**2**_ **F**_**1**_ **(p**_**1**_ **)/F(p**_**1**_ **, p**_**2**_ **)}, with F**_**1**_ **(p**_**1**_ **) and F**_**2**_ **(p**_**2**_ **) the marginal non-null cdfs of SNPs for SCZ and BD, respectively.** For enriched samples, p-values will tend to be smaller than predicted from the uniform distribution, so that F_1_(p_1_) **≥** p_1_ and F_2_(p_2_) **≥** p_2_. **Then**

$$\begin{array}{l} \max\left\{p_{1}F_{2}\left(p_{2}\right)/F\left(p_{1},\;p_{2}\right),\;p_{2}F_{1}\left(p_{1}\right)/F\left(p_{1},\;p_{2}\right)\right\}\\ \begin{array}{l} \geq \left[\pi_{0}+\pi_{1}+\pi_{2}\right]\;\max\left\{p_{1}F_{2}\left(p_{2}\right)/F\left(p_{1},\;p_{2}\right),\;p_{2}F_{1}\left(p_{1}\right)/F\left(p_{1},\;p_{2}\right)\right\}\\ \geq \left[\pi_{0}p_{1}p_{2}+\pi_{1}p_{2}F_{1}\left(p_{1}\right)+\pi_{2}p_{1}F_{2}\left(p_{2}\right)\right]/F\left(p_{1},\;p_{2}\right). \end{array} \end{array}$$

**Under the assumption that SNPs are independent if one or both are null, reasonable for disjoint samples, this last quantity is precisely the conjunction FDR given in Eq (6)**. Thus, Eq (7) is a conservative model-free estimate of the conjunction FDR. We present a complementary model-based approach to estimating conjunction FDR in the S1 Text.

We assigned the conjunction FDR values by interpolation into a bi-directional two-dimensional look-up table (S3 Fig). All SNPs with conjunction FDR<0.05 (−log_10_(FDR)>1.3) with schizophrenia and bipolar disorder considered jointly are listed in Table 3 (after pruning), together with the corresponding z-scores and minor alleles. The z-scores were calculated from the p-values and the direction of effect was determined by the risk allele.

**Table 3:** Conjunction FDR; pleiotropic loci in SCZ and BD (SCZ&BD).

locus	SNP	neighbor gene	Chr	A1	A2	conjfdr BD&SCZ	z-score BD	z-score SCZ
1	rs2252865	*RERE*	*1p36*.*23*	T	C	0.030	3.696	3.494
2	rs4650608	*IFI44*	*1p31*.*1*	T	C	0.043	3.289	3.711
4	rs11205362	*PRP3*	*1q21*.*1*	G	A	0.033	3.404	3.262
8	rs9834970	*TRANK1*	*3p22*.*2*	C	T	0.027	3.470	3.965
9	rs4687657	*ITIH4†*	*3p21*.*1*	G	T	0.028	3.787	3.781
11	rs3134942	*NOTCH4†*	*6p21*.*3*	G	T	0.048	3.251	3.571
15	rs3757440	*MAD1L1*	*7p22*	A	G	0.031	3.490	3.425
20	rs10883757	*TRIM8*	*10q24*.*3*	C	T	0.040	3.261	3.046
22	rs1006737	*CACNA1C†*	*12p13*.*3*	A	G	0.022	4.553	4.137
26	rs961196	*TTC7B*	*14q32*.*11*	C	T	0.044	3.618	2.960
28	rs12708772	*SHISA9*	*16p13*.*12*	C	T	0.044	3.294	2.955
31	rs1800359	*ZNF276*	*16q24*.*3*	A	G	0.035	3.329	3.165
33	rs159788	*BC039673*	*20p13*	G	A	0.034	3.411	-3.232
35	rs381523	*PPM1F*	*22q11*.*22*	A	G	0.045	3.220	3.166

Independent complex or single gene loci (r^2^ < 0.2) with SNP(s) with a conjunctional FDR (conjFDR) < 0.05 in schizophrenia (SCZ) *and* bipolar disorder (BD). All SNPs with a conjFDR value < 0.05 (bidirectional association, i.e. association with SCZ given association with BD (condFDR< 0.05) and association with BD given association with SCZ (condFDR<0.05)) are listed and sorted in each LD block. We defined the most significant SNP in each LD block based on the minimum conjFDR. All independent loci are listed consecutively, and the same locus number are used as in the condFDR < 0.05 results (Table 1). Chromosome (Chr). Z-scores for each pleiotropic locus are provided, with minor allele (A1) and major allele (A2). All data were first corrected for genomic inflation. †Same locus identified in previous BD or SCZ genome-wide association studies.

**Incorrect mathematical definition of the conjunction FDR in the ‘Conditional and Conjunction Local False Discovery Rate’ section of the S1 Text**.

There are also errors under the sub-heading ‘Conditional and Conjunction Local False Discovery Rate’ in the S1 Text. Please view the correct S1 Text here, with updates to the text in red.

## Supporting Information

S3 Fig Conjunction FDR bi-directional 2-D Look-up table. (DOC)

S1 Text Supporting statistical methods. (DOC)
