# Correction: Correction: Improved Detection of Common Variants Associated with Schizophrenia and Bipolar Disorder Using Pleiotropy-Informed Conditional False Discovery Rate

**DOI:** 10.1371/journal.pgen.1005859

**Published:** 2016-02-05

**Authors:** The PLOS Genetics Staff

## Notice of Republication

This correction notice was republished on December 1, 2015, to correct hyperlinking errors which were introduced during the production process.

In the Supporting Information, the legend for S3 Fig was hyperlinked to the S1 Text document and vice versa. The publisher apologizes for the errors.
