# Supplementary material for: Improved Detection of Common Variants Associated with Schizophrenia and Bipolar Disorder Using Pleiotropy-Informed Conditional False Discovery Rate
Source: PLoS Genet. 2013 Apr 25;9(4):e1003455. doi: 10.1371/journal.pgen.1003455 (PMC3636100; doi:10.1371/journal.pgen.1003455)
Supplement: Figure S1 — Stratified Q-Q plots of nominal versus empirical -log10 p-values of genic vs. intergenic regions, controlling for genomic inflation in schizophrenia (p<5×10^−8^). The plots illustrate the enrichment of genic SNPs compared to all SNPs, and lack of enrichment for intergenic SNPs. The plot in top panel is based on uncorrected data, showing inflation (λ 1.24). The intergenic SNPs were used for genomic control. The plot in bottom panel is based on data after correcting for inflation. (DOC) [file pgen.1003455.s001.doc]

**Figure S1. Stratified Q-Q plots for genomic control based on intergenic SNPs**
