# Supplementary material for: Improved Detection of Common Variants Associated with Schizophrenia and Bipolar Disorder Using Pleiotropy-Informed Conditional False Discovery Rate
Source: PLoS Genet. 2013 Apr 25;9(4):e1003455. doi: 10.1371/journal.pgen.1003455 (PMC3636100; doi:10.1371/journal.pgen.1003455)
Supplement: Figure S2 — Based on the combination of p-value for the SNP in schizophrenia (SCZ) and bipolar disorder (BD), we assigned a conditional FDR value for SCZ to each SNP, by interpolation into a 2-dimensional lookup table. This is shown in upper panel for SCZ conditioned on BD, denoted FDRSCZ | BD. BD conditioned on SCZ, denoted FDRBD|SCZ is shown in lower panel. Color scale refers to FDR values. (DOC) [file pgen.1003455.s002.doc]

**Figure S2. Conditional FDR 2-D Look-up tables**

Empirical –log_10_p values
