# Supplementary material for: Improved Detection of Common Variants Associated with Schizophrenia and Bipolar Disorder Using Pleiotropy-Informed Conditional False Discovery Rate
Source: PLoS Genet. 2013 Apr 25;9(4):e1003455. doi: 10.1371/journal.pgen.1003455 (PMC3636100; doi:10.1371/journal.pgen.1003455)
Supplement: Figure S3 — Based on the combination of p-value for the SNP in schizophrenia (SCZ) given bipolar disorder (BD), and the combination of p-value for the SNP in BD given SCZ, we assigned a conjunction FDR value for SCZ and BD to each SNP, by interpolation into a bi-directional 2-D look up table. This is denoted FDRBD&SCZ. Color scale refers to FDR values. (DOC) [file pgen.1003455.s003.doc]

**Figure S3. Conjunction FDR bi-directional 2-D Look-up table**
