# Supplementary figures and images for: Improved Detection of Common Variants Associated with Schizophrenia and Bipolar Disorder Using Pleiotropy-Informed Conditional False Discovery Rate
Source: PLoS Genet. 2013 Apr 25;9(4):e1003455. doi: 10.1371/journal.pgen.1003455 (PMC3636100; doi:10.1371/journal.pgen.1003455)

**Figure S4. Empirical and model-based QQ-plots for schizophrenia**

**
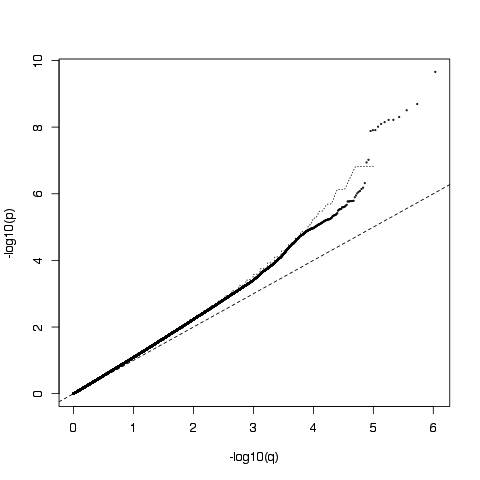
**

Supplement: Figure S4 — QQ-plot for schizophrenia (SCZ). Dotted lines give marginal fit based on MCMC estimates for the two-groups mixture model. Dashed line gives null distribution. (DOC) [file pgen.1003455.s004.doc]

**Figure S5. Empirical and model-based QQ-plots for bipolar disorder**


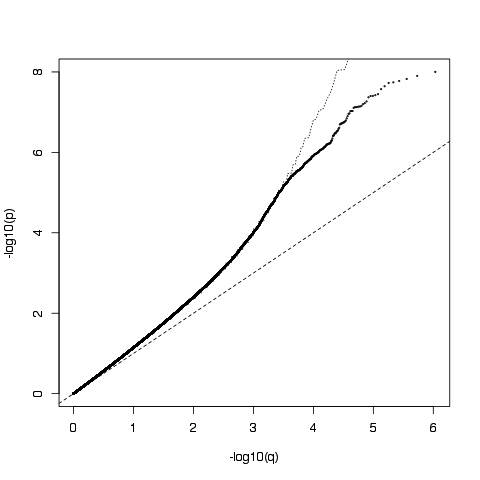

Supplement: Figure S5 — QQ-plot for bipolar disorder (BD). Dotted lines give marginal fit based on MCMC estimates for the two-groups mixture model. Dashed line gives null distribution. (DOC) [file pgen.1003455.s005.doc]

**Figure S6. Empirical and model-based QQ-plots for type 2 diabetes**


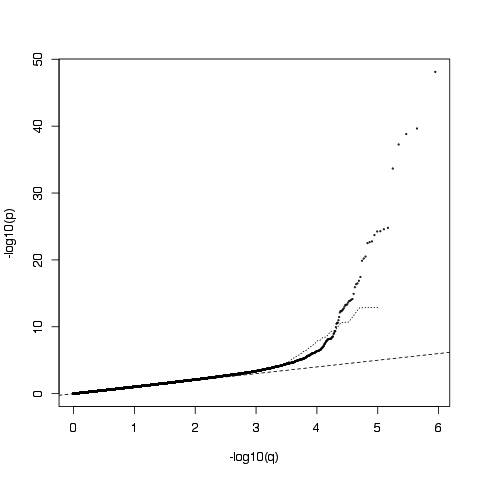

Supplement: Figure S6 — QQ-plot for type 2 diabetes (T2D). Dotted lines give marginal fit based on MCMC estimates for the two-groups mixture model. Dashed line gives null distribution. (DOC) [file pgen.1003455.s006.doc]
