# Supplementary material for: Improved Detection of Common Variants Associated with Schizophrenia and Bipolar Disorder Using Pleiotropy-Informed Conditional False Discovery Rate
Source: PLoS Genet. 2013 Apr 25;9(4):e1003455. doi: 10.1371/journal.pgen.1003455 (PMC3636100; doi:10.1371/journal.pgen.1003455)
Supplement: Figure S7 — ROC curves for schizophrenia. Solid black line shows the proportion of non-null SNPs declared significant (Power; Sensitivity) for a given local false discovery rate (Local fdr; 1-specificity). The corresponding ROC curve for schizophrenia (SCZ) local false discovery rate conditional on type 2 diabetes (T2D) is given in red (SCZ | T2D). Red and black lines are nearly overlapping due to almost no increase in power. Power resulting from a hypothetical doubling of effective subject sample size is given by the dashed black line. (DOC) [file pgen.1003455.s007.doc]

**Figure S7. ROC power curve for SCZ and SCZ | T2D**
