# Supplementary material for: Improved Detection of Common Variants Associated with Schizophrenia and Bipolar Disorder Using Pleiotropy-Informed Conditional False Discovery Rate
Source: PLoS Genet. 2013 Apr 25;9(4):e1003455. doi: 10.1371/journal.pgen.1003455 (PMC3636100; doi:10.1371/journal.pgen.1003455)
Supplement: Table S1 — Associated loci and previous GWAS findings. (DOC) [file pgen.1003455.s008.doc]

**Table S1. Associated loci and previous GWAS findings**

| **SCZ|BD** | | | |
| --- | --- | --- | --- |
| **Gene** | **Chr. loc.** | **Name encoded protein** | **Association SCZ,BD (PheGenI)** |
| *RERE* | 1p36.23 | arginine-glutamic acid dipeptide (RE) repeats | SCZ^1^ (Borderline) |
| *KIAA1026* | 1p36.21 | (similar to)kazrin, periplakin interacting protein |  |
| *BC042538* | 1p35.2 | | |
| *IFI44* | 1p31.1 | interferon-induced protein 44 |  |
| *LPAR3* | 1p22.3 | lysophosphatidic acid receptor 3 |  |
| *AK094607* | 1p21.3 | MIR137 host gene (non-protein coding) | SCZ^1^ (After replication) |
| *PRP3* | 1q21.1 | PRP3 pre-mRNA processing factor 3 homolog |  |
| *RAD51AP2* | 2p24.2 | RAD51 associated protein 2 |  |
| *GCKR* | 2p23 | glucokinase (hexokinase 4) regulator |  |
| *VRK2* | 2p16.1 | vaccinia related kinase 2 | SCZ^2^ |
| *SH3RF3* | 2q13 | SH3 domain containing ring finger 3 |  |
| *KIF5C* | 2q23.1 | kinesin family member 5C |  |
| *CWC22* | 2q31.3 | CWC22 spliceosome-associated protein homolog |  |
| *PCGEM1* | 2q32 | rostate-specific transcript 1 (non-protein coding) |  |
| *C2orf82* | 2q37.1 | chromosome 2 open reading frame 82 |  |
| *AGAP1* | 2q37 | ArfGAP with GTPase domain, ankyrin repeat and PH domain 1 | SCZ^1^ (Borderline) |
| *TRANK1* | 3p22.2 | tetratricopeptide repeat and ankyrin repeat containing 1 | BD^3^, BD^4^ (Borderline), SCZ ^1^(Borderline) |
| *ITIH4* | 3p21.1 | inter-alpha-trypsin inhibitor heavy chain family, member 4 | SCZ^1^ (After combining with BD) |
| *PTPRG* | 3p21-p14 | protein tyrosine phosphatase, receptor type, G |  |
| *DKFZp434A128* | 3q26.33 | | |
| *SORBS2* | 4q35.1 | sorbin and SH3 domain containing 2 |  |
| *SCGN* | 6p22.3-p22.1 | secretagogin, EF-hand calcium binding protein |  |
| *HIST1H2BC* | 6p22.1 | histone cluster 1, H2bc |  |
| *HIST1H2BD* | 6p21.3 | histone cluster 1, H2bd |  |
| *BC035101* | 6p22.1 | uncharacterized LOC100507173 |  |
| *ZSCAN23* | 6p22.1 | zinc finger and SCAN domain containing 23 |  |
| *ZNF311* | 6p22.1 | zinc finger protein 311 |  |
| *TRIM26* | 6p21.32-p22.1 | tripartite motif containing 26 | SCZ^1^ |
| *MICA* | 6p21.33 | MHC class I polypeptide-related sequence A |  |
| *HCP5* | 6p21.3 | HLA complex P5 (non-protein coding) |  |
| *LY6G6C* | 6p21.33 | lymphocyte antigen 6 complex, locus G6C |  |
| *HLA-DRA* | 6p21.3 | major histocompatibility complex, class II, DR alpha |  |
| *HLA-DRB5* | 6p21.3 | major histocompatibility complex, class II, DR beta 5 |  |
| *HLA-DQB2* | 6p21 | major histocompatibility complex, class II, DQ beta 2 |  |
| *HLA-DOB* | 6p21.3 | major histocompatibility complex, class II, DO beta |  |
| *HLA-DMA* | 6p21.3 | major histocompatibility complex, class II, DM alpha |  |
| *CUL9* | 6p21.1 | cullin 9 |  |
| *FTSJ2* | 7p22 | FtsJ RNA methyltransferase homolog 2 |  |
| *AK055863* | 8p23.1 | | |
| *MMP16* | 8q21.3 | matrix metallopeptidase 16 | SCZ^1^ (After replication) |
| *ABCA1* | 9q31.1 | ATP-binding cassette, sub-family A (ABC1), member 1 |  |
| *AK094154* | 10p14 | | |
| *ANK3* | 10q21 | ankyrin 3, node of Ranvier (ankyrin G) | BD^3, 5,6^, BD^4^ (Border-line), SCZ^1^ (After combining with BD), SCZ^7^(Borderline) |
| *RRP12* | 10q24.1 | ribosomal RNA processing 12 homolog |  |
| *SUFU* | 10q24.32 | suppressor of fused homolog |  |
| *CNNM2* | 10q24.32 | cyclin M2 | SCZ^1^ (After replication) |
| *NT5C2* | 10q24.32 | 5'-nucleotidase, cytosolic II | SCZ^1^ (After replication) |
| *PIK3C2A* | 11p15.5-p14 | phosphatidylinositol-4-phosphate 3-kinase, catalytic subunit type 2 alpha | SCZ^1^ (Borderline) |
| *LRP5* | 11q13.4 | low density lipoprotein receptor-related protein 5 |  |
| *GAL* | 11q13.3 | galanin prepropeptide |  |
| *LOC399959* | 11q24.1 | mir-100-let-7a-2 cluster host gene (non-protein coding) |  |
| *SNX19* | 11q25 | sorting nexin 19 | SCZ^1^ (Borderline) |
| *IGSF9B* | 11q25 | immunoglobulin superfamily, member 9B |  |
| *CACNA1C* | 12p13.3 | calcium channel, voltage-dependent, L type, alpha 1C subunit | SCZ^1^ (After combining with BD), BD^4,5,6^ |
| *DAOA* | 13q34 | D-amino acid oxidase activator | SCZ^8^ (Borderline) |
| *TTC7B* | 14q32.11 | tetratricopeptide repeat domain 7B |  |
| *TMCO5A* | 15q14 | transmembrane and coiled-coil domains 5A | BD^5^ (Borderline) |
| *C15orf54* | 15q14 | chromosome 15 open reading frame 54 | BD^5^ (Borderline) |
| *PLCB2* | 15q15 | phospholipase C, beta 2 | SCZ^1^ (Borderline) |
| *BC033962* | 15q22.2 | | |
| *NMB* | 15q22-qter | neuromedin B |  |
| *NTRK3* | 15q25 | neurotrophic tyrosine kinase, receptor, type 3 |  |
| *DNAJA3* | 16p13.3 | DnaJ (Hsp40) homolog, subfamily A, member 3 |  |
| *SHISA9* | 16p13.12 | shisa homolog 9 | SCZ ^9^(Borderline) |
| *ZNF276* | 16q24.3 | zinc finger protein 276 |  |
| *AK093940* | 18q21.2 | | |
| *BC039673* | 20p13 | | |
| *PPM1F* | 22q11.22 | protein phosphatase, Mg2+/Mn2+ dependent, 1F |  |
| *LARGE* | 22q12.3 | like-glycosyltransferase |  |
| *EP300* | 22q13.2 | E1A binding protein p300 |  |
| *RPL23AP82* | 22q13.33 | ribosomal protein L23a pseudogene 82 |  |

| ***BD|SCZ (not already in SCZ|BD part of Table above)*** | | | |
| --- | --- | --- | --- |
| **Gene** | **Chr. loc.** | **Name encoded protein** | **Association SCZ,BD (PheGenI)** |
| *NGF* | 1p13.1 | nerve growth factor (beta polypeptide) |  |
| *PLEKHO1* | 1q21.2 | pleckstrin homology domain containing, family O member 1 |  |
| *SIPA1L2* | 1q42.2 | signal-induced proliferation-associated 1 like 2 |  |
| *FLJ16124* | 2p14 | FLJ16124 protein |  |
| *LMAN2L* | 2q11.2 | lectin, mannose-binding 2-like | BD ^3^, BD^,4,5^(Borderline) |
| *ITIH3* | 3p21.1 | inter-alpha-trypsin inhibitor heavy chain 3 | BD ^4^ (After combining with SCZ) |
| *ODZ2* | 5q34 | odz, odd Oz/ten-m homolog 2 |  |
| *NOTCH4* | 6p21.3 | notch 4 | SCZ ^2^ |
| *SYNE1* | 6q25 | spectrin repeat containing, nuclear envelope 1 | BD ^4,5^ (Borderline), BD ^6,10,11^ |
| *RPS6KA2* | 6q27 | ribosomal protein S6 kinase, 90kDa, polypeptide 2 |  |
| *MAD1* | MAD1L1 | | |
| *MAD1L1* | 7p22 | MAD1 mitotic arrest deficient-like 1 | SCZ^8^ (Borderline), BD ^4^, ^8^ (Borderline) |
| *THSD7A* | 7p21.3 | thrombospondin, type I, domain containing 7A |  |
| *AX747593* | 8q13.2 | | |
| *CACNB2* | 10p12 | calcium channel, voltage-dependent, beta 2 subunit |  |
| *TRIM8* | 10q24.3 | tripartite motif containing 8 |  |
| *ODZ4* | 11q14.1 | odz, odd Oz/ten-m homolog 4 | BD ^4^(After replication) |
| *DHH* | 12q13.1 | desert hedgehog |  |
| *NEDD1* | 12q23.1 | neural precursor cell expressed, developmentally down-regulated 1 |  |
| *SLITRK1* | 13q31.1 | SLIT and NTRK-like family, member 1 |  |
| *EML1* | 14q32 | echinoderm microtubule associated protein like 1 |  |
| *AKTIP* | 16q12.2 | AKT interacting protein |  |
| *CDH11* | 16q21 | cadherin 11, type 2, OB-cadherin |  |
| *C16orf7* | 16q24 | chromosome 16 open reading frame |  |
| *RASIP1* | 19q13.33 | Ras interacting protein 1 | BD ^4^(borderline) |
| *BC039673* | 20p13 | | |
| *ITGB2* | 21q22.3 | integrin, beta 2 (complement component 3 receptor 3 and 4 subunit) |  |

BD = bipolar disorder, SCZ = schizophrenia. ‘Borderline’ indicates not genome-wide significant p-values. ‘After replication’ indicates findings in original GWAS of SCZ or BD (used in the current study) that were not genome-wide significant, but reached significance only after including a large replication sample (see ref 1 and 4 for details). Some of the findings in Ripke et al (ref 1) were not significant after GC correction. PheGenI data base and PubMed searches were used to identify previous results.

1. Ripke S, Sanders AR, Kendler KS, Levinson DF, Sklar P, et al; Schizophrenia Psychiatric Genome-Wide Association Study (GWAS) Consortium. Genome-wide association study identifies five new schizophrenia loci. Nat Genet. 2011 Sep 18;43(10):969-76. doi: 10.1038/ng.940.
2. Stefansson H, Ophoff RA, Steinberg S, Andreassen OA, Cichon S, et al. Common variants conferring risk of schizophrenia. Nature. 2009 Aug 6;460(7256):744-7. Epub 2009 Jul 1.
3. Chen DT, Jiang X, Akula N, Shugart YY, Wendland JR, Steele CJ, Kassem L, Park JH, Chatterjee N, Jamain S, Cheng A, Leboyer M, Muglia P, Schulze TG, Cichon S, Nöthen MM, Rietschel M; BiGS, McMahon FJ. Genome-wide association study meta-analysis of European and Asian-ancestry samples identifies three novel loci associated with bipolar disorder. Mol Psychiatry. 2011 Dec 20. doi: 10.1038/mp.2011.157.
4. Sklar P, Ripke S, Scott LJ, Andreassen OA, Cichon S, et al; Psychiatric GWAS Consortium Bipolar Disorder Working Group. Large-scale genome-wide association analysis of bipolar disorder identifies a new susceptibility locus near ODZ4. Nat Genet. 2011 Sep 18;43(10):977-83. doi: 10.1038/ng.943.
5. Ferreira MA, O'Donovan MC, Meng YA, Jones IR, Ruderfer DM, et al. Wellcome Trust Case Control Consortium. Collaborative genome-wide association analysis supports a role for ANK3 and CACNA1C in bipolar disorder. Nat Genet. 2008 Sep;40(9):1056-8.
6. Liu Y, Blackwood DH, Caesar S, de Geus EJ, Farmer A, et al.; Wellcome Trust Case-Control Consortium. Meta-analysis of genome-wide association data of bipolar disorder and major depressive disorder. Mol Psychiatry. 2011 Jan;16(1):2-4. Epub 2010 Mar 30.
7. Athanasiu L, Mattingsdal M, Kähler AK, Brown A, Gustafsson O, et al. Gene variants associated with schizophrenia in a Norwegian genome-wide study are replicated in a large European cohort. J Psychiatr Res. 2010 Sep;44(12):748-53.
8. Wang KS, Liu XF, Aragam N. A genome-wide meta-analysis identifies novel loci associated with schizophrenia and bipolar disorder. Schizophr Res. 2010 Dec;124(1-3):192-9.
9. O'Donovan MC, Craddock N, Norton N, Williams H, Peirce et al. Molecular Genetics of Schizophrenia Collaboration. Identification of loci associated with schizophrenia by genome-wide association and follow-up. Nat Genet. 2008 Sep;40(9):1053-5.
10. Smith EN, Koller DL, Panganiban C, Szelinger S, Zhang P, et al. Genome-wide association of bipolar disorder suggests an enrichment of replicable associations in regions near genes. PLoS Genet. 2011 Jun;7(6):e1002134. Epub 2011 Jun 30.
11. Green EK, Grozeva D, Forty L, Gordon-Smith K, Russell E, et al. Association at SYNE1 in both bipolar disorder and recurrent major depression. Mol Psychiatry. 2012 May 8. doi: 10.1038/mp.2012.48. [Epub ahead of print]
