# Supplementary material for: Correction: Improved Detection of Common Variants Associated with Schizophrenia and Bipolar Disorder Using Pleiotropy-Informed Conditional False Discovery Rate
Source: PLoS Genet. 2015 Nov 5;11(11):e1005544. doi: 10.1371/journal.pgen.1005544 (PMC4634985; doi:10.1371/journal.pgen.1005544)
Supplement: S1 Text — (DOC) [file pgen.1005544.s002.doc]

**S1 Text Supporting statistical methods**

**Four-Groups Mixture Model**

Here, we describe the model-based methodology for computing pleitropy-informed conditional and conjunction analyses, complementary to the model-free approach presented in the main text. Let z be the GWAS test statistic (z-score) with corresponding nominal significance p (two-tailed probability of observed z-score under the null hypothesis of no effect). A standard Bayesian *two-groups mixture model* is given by

f(z) = π_0_f_0_(z) + (1-π_0_)f_1_(z) [S1]

where f_0_ is the null distribution (e.g., standard normal after appropriate genomic control), f_1_ is the non-null distribution (which may be estimated parametrically or non-parametrically, and π_0_ is the proportion of null SNPs. For example, Yang et al. formulate [S1] as a mixture of Gaussian distributions. From model [S1] we can define the Bayesian False Discovery Rate (denoted by FDR) and the local False Discovery Rate (denoted by fdr) for a given effect size z as

FDR(z) = π_0_F_0_(z)/ F(z) [S2]

fdr(z) = π_0_f_0_(z)/ f(z) [S3]

where F_0_(z) and F(z) are the cumulative distribution functions (cdfs) corresponding to f_0_(z) and f(z), respectively. In what follows we focus on extensions to conditional and conjunction fdr (Eq. [S3]); it is straightforward to extend this to include conditional and conjunction FDR (Eq. [S2]).

We generalize Eq. [S1] to bivariate z-scores from two phenotypes (z_1_ for phenotype 1 and z_2_ for phenotype 2) using a bivariate density from a *four-groups mixture model*

f(z_1_, z_2_) = π_0_f_0_(z_1_, z_2_) + π_1_f_1_(z_1_, z_2_) + π_2_f_2_(z_1_, z_2_) + π_3_f_3_(z_1_, z_2_) [S4]

where π_0_ is the proportion of SNPs for which both phenotypes are null, π_1_ is the proportion of SNPs where phenotype 1 is non-null and phenotype 2 is null, π_2_ is the proportion of SNPs where phenotype 1 is null and phenotype 2 is non-null, and π_3_ is the proportion of SNPs where both phenotypes are non-null (i.e., the pleiotropic SNPs). The mixture densities in [S4] are given by

f_o_(z_1_, z_2_) = N(**0**,Σ_0_)

f_1_(z_1_, z_2_) = N(**0**,Σ_0_ + D_1_Σ_1_ D_1_^T^)

f_2_(z_1_, z_2_) = N(**0**,Σ_0_ + D_2_Σ_1_ D_2_^T^)

f_3_(z_1_, z_2_) = N(**0**,Σ_0_ + Σ_1_) [S5]

where Σ_0_ and Σ_1_ are arbitrary 2 x 2 variance-covariance matrices, D_1_ is a 2 x 2 matrix with a one in the first row, first column and zeros elsewhere, and D_2_ is a 2 x 2 matrix with a one in the second row, second column and zeros elsewhere. This model is a simple bivariate extension of the mixture of Gaussians employed in Yang et al.. An alternative approach could model the non-null marginal densities via nonparametric methods and model their dependence using a copula formulation. Note, model [S5] allows for empirical null distributions with marginal variances, the diagonal elements of Σ_0_, potentially larger than 1. Moreover, the off-diagonal elements of Σ_0_ allow for non-zero covariance between null SNPs. The proportions π = (π_0_, π_1_, π_2_, π_3_) and the parameters of the non-null distributions can be estimated using methods such as Markov Chain Monte Carlo (MCMC) algorithms or maximum likelihood (ML) estimation. Figures S4 and S5, present empirical and MCMC-estimated marginal and conditional QQ-plots for SCZ and BD, respectively. To provide a comparison of a trait only weakly pleiotropic with SCZ and BD, we show the empirical and model-based conditional QQ-plots of SCZ given level of significance of type 2 diabetes (T2D) GWAS data in Figure S6. QQ-plots of BD conditional on T2D, not shown here, are qualitatively similar.

The estimated vector of probabilities π = (π_0_, π_1_, π_2_, π_3_) from these fits can be used to test whether the degree of pleiotropy is significantly higher than expected by chance if both phenotypes were independent. Independence implies that the joint pdf of both phenotype summary scores is a product of two two-group mixture models (two independent versions of Eq. [S1]). It is easy to show that testing for excess pleiotropy over that predicted by independence is equivalent to showing that π_3_ > π_1_π_2_/π_0_ in Eq. [S4] or equivalently that the log-odds ratio

LOR(Phen. 1, Phen. 2) = log{π_3_/1-π_3_} – log{(π_1_π_2_/π_0_)/(1- π_1_π_2_/π_0_)} [S6]

is greater than zero. Using posterior draws from the MCMC algorithm, estimates of LOR with 95% posterior credible intervals are: LOR(SCZ,BD) = 7.1 [8.0, 8.9], LOR(SCZ,T2D) = 1.2 [-0.6, 3.1], and LOR(BD,T2D) = 3.8 [3.1, 4.5]. In particular, the departure from independence of SCZ and BD is highly significant, with a 95% CI bounded well above zero, whereas pleiotropy between SCZ and T2D is not significantly different from zero. Estimates and 95% CIs were produced using the SCZ/BD data z scores estimated using non-overlapping controls, and include an adjustment to account for correlation of SNPs (i.e., LD) that assumes an effective degree of freedom of 500,000 independent SNPs.

We also estimated the proportion of pleiotropic SNPs for each phenotype. For example, π_3_/(π_1_ + π_3_) is the proportion of pleiotropic SNPs for phenotype 1 (i.e., the proportion of non-null SNPs for phenotype 1 that are also non-null for phenotype 2). Again using the MCMC estimates from the bivariate mixture of Gaussians, the proportion of pleiotropic SNPs for BD with SCZ was 0.78 (95% CI: [0.68, 0.88]), the proportion for SCZ with BD was 0.90 [0.78, 1.00], the proportion for SCZ with T2D was 0.02 [0.0, 0.09], the proportion for BD with T2D was 0.09 [0.01, 0.16]. ML estimates and 95% CIs were again produced using the SCZ/BD data z-score estimates with non-overlapping controls, and include an adjustment to account for correlation of SNPs. The large increase in power for BD | SCZ noted below is due in part to the high proportion of non-null SCZ SNPs that are also non-null BD SNPs.

**Conditional and Conjunction Local False Discovery Rate**

From the estimates of the four-groups mixture pdf (Eq. [S4]) we can compute estimates of the conditional pdf of z_1_ given z_2_ and hence the *conditional fdr* of the first phenotype given the second

fdr(z_1_| z_2_) = f(z_1_| z_1_ null, z_2_) Pr(z_1_ null | z_2_) / f(z_1_| z_2_) [S6]

where f(z_1_| z_1_ null, z_2_) is the null density of z_1_ conditional on z_2_, Pr(z_1_ null | z_2_) is the probability that z_1_ is null given z_2_, and f(z_1_| z_2_) is the mixture density of z_1_ conditional on z_2_. With component densities as given in Eq. [S5], this becomes

fdr(z_1_| z_2_) = [π_0_ f_0_(z_1_, z_2_) + π_2_ f_2_(z_1_, z_2_)]/ f(z_1_, z_2_), [S7]

where f(z_1_, z_2_) is the joint density given in Eq. [S4].

We can also compute the conjunctional fdr of both phenotypes as

fdr(z_1_, z_2_) = [f(z_1_, z_2_| z_1_ null, z_2_ null) Pr(z_1_ null, z_2_ null)

+ f(z_1_, z_2_| z_1_ non-null, z_2_ null) Pr(z_1_ non-null, z_2_ null)

+ f(z_1_, z_2_| z_1_ null, z_2_ non-null) Pr(z_1_ null, z_2_ non-null)]/ f(z_1_, z_2_)

[S8]

With densities given in Eq. [S5], this becomes

fdr(z_1_, z_2_) = [π_0_ f_0_(z_1_, z_2_)+ π_1_ f_1_(z_1_, z_2_)+ π_2_ f_2_(z_1_, z_2_)]/ f(z_1_, z_2_) [S9]

**Conditional Local False Discovery Rate and Power**

Conditional local false discovery rates fdr(z_1_| z_2_) can lead to significant increases in power when two phenotypes are genuinely pleiotropic (i.e., when LOR(Phen. 1, Phen. 2) is significantly larger than zero). Here, power is defined in terms of the probability of rejecting the null hypothesis for SNPs that are in fact non-null for a given fdr threshold α. In this sense power corresponds to sensitivity to detect non-null SNPs and power diagnostics correspond can be presented as ROC-type curves as detailed in Efron. In Figures 5, 6 and S7 we present power diagnostic plots for conditional fdr estimated using the MCMC estimates from the bivariate mixture of Gaussians model. The x-axis is the fdr (1-specificity) whereas the y-axis is the proportion of non-null SNPs rejected (sensitivity, or power). ROC curves include marginal fdrs and conditional fdrs of phenotype 1 given phenotype 2. In particular these plots demonstrate a very large increase in power for using fdr of BD | SCZ. For comparison, we include ROC curves from a hypothetical doubling of the sample size (corresponding to scaling the variance-covariance matrix Σ_1_ of the non-null distribution by two.

Note, estimates of power in the sense described above are sensitive to assumptions about the shape of the non-null distribution near zero. However, *relative power* (the ratio of sensitivity of conditional fdr with marginal fdr for a given threshold α) is well identified. For example, using the fdr cut-off α ≤ .05, the ratio of power for conditional fdr of BD | SCZ vs. marginal fdr of BD is ~15. The ratio of power for unconditional vs. conditional fdr for SCZ | BD is 5.7. In contrast, the ratio of power for unconditional vs. conditional fdr for SCZ | T2D is 1.00, indicating no improvement whatsoever.

**Q-Q plots**

Q-Q plots compare a nominal probability distribution against an empirical distribution. In the presence of all null relationships, nominal p-values form a straight line on a Q-Q plot when plotted against the empirical distribution. For each phenotype, for all SNPs and for each conditional subset, -log_10_ nominal p-values were plotted against -log_10_ empirical p-values. Leftward deflections of the observed distribution from the projected null line reflect increased tail probabilities in the distribution of test statistics (z-scores) and consequently an over-abundance of low p-values compared to that expected by chance under the global null hypothesis, also termed ‘enrichment’.

**Supporting References**

1. Efron B (2010) Large-scale inference : empirical Bayes methods for estimation, testing, and prediction. Cambridge ; New York: Cambridge University Press. xii, 263 p. p.

2. Yang J, Weedon MN, Purcell S, Lettre G, Estrada K, et al. (2011) Genomic inflation factors under polygenic inheritance. Eur J Hum Genet 19: 807-812.

3. Joe H (1997) Multivariate models and multivariate dependence concepts: Chapman & Hall/CRC.

4. Voight BF, Scott LJ, Steinthorsdottir V, Morris AP, Dina C, et al. (2010) Twelve type 2 diabetes susceptibility loci identified through large-scale association analysis. Nat Genet 42: 579-589.

5. Efron B (2007) Size, power and false discovery rates. The Annals of Statistics 35: 1351–1377.
